# Intramolecular Hydrogen Bonding in N^6^-Substituted 2-Chloroadenosines: Evidence from NMR Spectroscopy

**DOI:** 10.3390/ijms24119697

**Published:** 2023-06-02

**Authors:** Maria Ya. Berzina, Barbara Z. Eletskaya, Alexei L. Kayushin, Elena V. Dorofeeva, Olga I. Lutonina, Ilya V. Fateev, Olga N. Zhavoronkova, Arthur R. Bashorin, Alexandra O. Arnautova, Olga S. Smirnova, Konstantin V. Antonov, Alexander S. Paramonov, Maxim A. Dubinnyi, Roman S. Esipov, Anatoly I. Miroshnikov, Irina D. Konstantinova

**Affiliations:** 1 Shemyakin and Ovchinnikov Institute of Bioorganic Chemistry, Russian Academy of Sciences, Miklukho-Maklaya St. 16/10, 117997 Moscow, Russiaantonov.kant@yandex.ru (K.V.A.);; 2 Department of Biological and Medical Physics, Moscow Institute of Physics and Technology (State University), 9 Institutskiy per., Dolgoprudny, 141700 Moscow, Russia

**Keywords:** intramolecular hydrogen bond, hindered rotation, 2D NMR, N^6^ adenosine derivatives

## Abstract

Two forms were found in the NMR spectra of N^6^-substituted 2-chloroadenosines. The proportion of the mini-form was 11–32% of the main form. It was characterized by a separate set of signals in COSY, ^15^N-HMBC and other NMR spectra. We assumed that the mini-form arises due to the formation of an intramolecular hydrogen bond between the N7 atom of purine and the N^6^–CH proton of the substituent. The ^1^H,^15^N-HMBC spectrum confirmed the presence of a hydrogen bond in the mini-form of the nucleoside and its absence in the main form. Compounds incapable of forming such a hydrogen bond were synthesized. In these compounds, either the N7 atom of the purine or the N^6^–CH proton of the substituent was absent. The mini-form was not found in the NMR spectra of these nucleosides, confirming the importance of the intramolecular hydrogen bond in its formation.

## 1. Introduction

Interest in the synthesis of new substituted adenosines remains high. Among these nucleosides, effective antimetabolites [1,2] and adenosine receptor agonists [3,4,5] have been found. NMR spectroscopy is a powerful tool for studying the adenosine derivatives. Among all NMR methods, two-dimensional heteronuclear NMR spectroscopy is of particular importance.

Substituents in the purine heterocycle can cause different forms to appear in the NMR spectra. Several theories have been put forward in the literature to explain the presence of different forms of adenine or adenosine derivatives.

**Figure 1:**
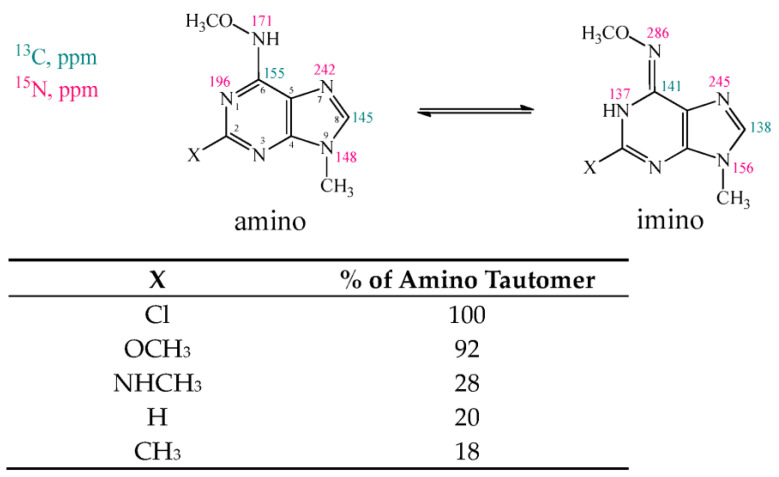
The amino and imino tautomers of 6-methoxyamino-purines (data from refs. [6,7,8]).

N^6^-substituted adenines and adenosine nucleosides are characterized by the phenomenon of amino–imino tautomerism (Figure 1) [6,7,8,9,10]. It has been reported that the amino form of adenine is absolutely dominant in solution; however, additional substitution can shift the tautomeric amino–imino equilibrium [11]. The introduction of an electron-withdrawing substituent at the C2 position of the purine shifts the equilibrium towards the amino form.

**Figure 2:**
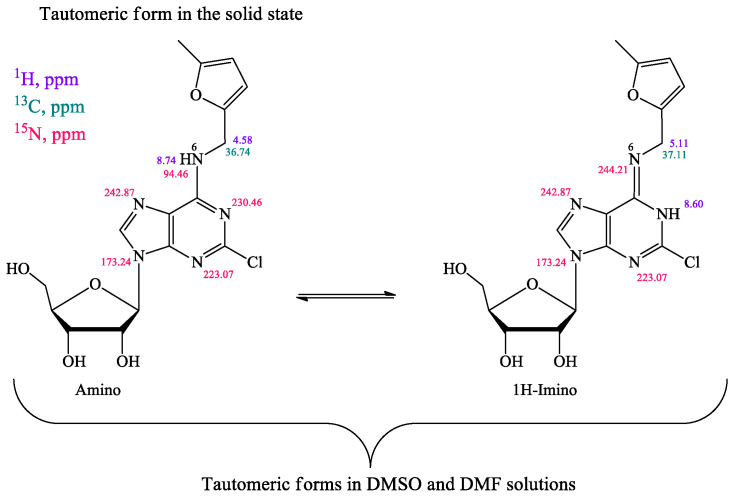
The proposed tautomeric forms of 2-chloro-N^6^-(5-methylfurfuryl)adenosine resulting from X-ray crystallographic and NMR spectroscopic studies (data from ref. [12]).

The presence of the second form in NMR was detected for kinetin and its derivatives [11,12]. Novotna et al. suggested that 2-chloro-N^6^-furfuryl-adenosine exists in two forms: amino and imino tautomers at room temperature in a solution. According to X-ray crystallography data, in the solid state only the amino form is presented [12] (Figure 2).

**Figure 3:**
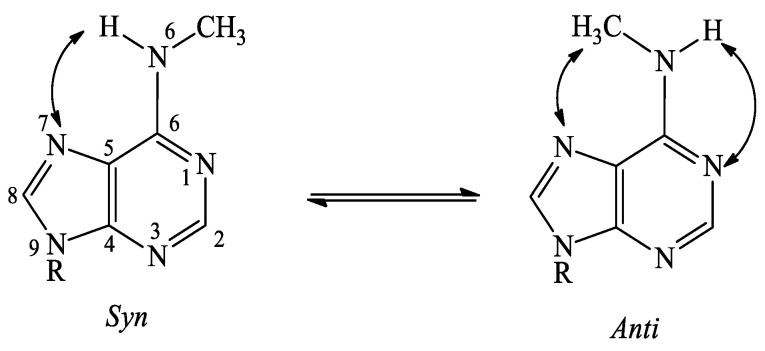
Proposed intramolecular hydrogen bonding in N^6^-substituted purines (data from refs. [14,15]).

Martin and Reese suggested that the second form in the NMR spectra emerged because of the hindered rotation. It was registered on cooling the 6-methylamino- and 6-dimethyladenosine solutions [13]. Engel et al. found that two distinct 6-methylamino isomers (rotamers) are associated with the *syn* and *anti* conformations of substituent group relative to the N1 of the purine cycle. The main form was *syn* (Figure 3) [14].

*Syn*-rotamer predominance was proposed to appear because of intramolecular hydrogen bonding between the hydrogen atom of the exocyclic amino group (N^6^) and N7 (Figure 3) [15]. Rotamer stabilization arises from two competing hydrogen bonds and a steric effect; *syn*-conformation is favored by N^6^–H∙∙∙N7 bonding, whereas the *anti*-form is stabilized by N^6^–H∙∙∙Nl bonding, but is destabilized by the steric interaction of the methyl group. 

The nature of the substituent at the C2 position affects the presence of the mini-form in NMR. In the NMR spectra of substituted adenosines, the second form is usually registered as broadened signals at N^6^H–CH [16,17,18,19,20], or not registered at all. The NMR spectra of the purines with an electron-withdrawing substituent (Cl, F) at position 2 show the presence of the second form at room temperature [11], and the authors often do not discuss the second form, but it can be seen in NMR spectra in the Supplementary Information [21,22,23,24,25,26]. Various C2-substituted purines with optically active substituents at C6 were obtained. The presence of the R or S enantiomers did not affect the amount of the second form [21].

At low temperatures (from 273 to 248 K), the mini-form appeared in both C2–H and C2–NH_2_-substituted purines [11]. NMR spectra are usually taken at 300 or 303 K, and the second form was not detected at such temperatures. In addition, the conditions for recording the spectra differed (CDCl_3_ instead of DMSO-*d*_6_, 100 °C rather than 30 °C) [25,27,28,29], and the relative amount of the mini-form was higher in DMSO than in CDCl_3_ [30]. In 7-deazakinetin, the second form did not appear in NMR even upon cooling [11]. The N7 atom made a key contribution to the restricted rotation around the C6–N^6^ bond. 

In summary, several models of the hindered rotation structure in C6-substituted purines and the related second form in NMR are described in the literature. 

We have recently synthesized a number of 2-chloradenosine analogs containing chiral amino acid amides and their analogs at the C6 position of the purine heterocycle [5]. When analyzing the NMR spectra of nucleosides, it was found that the substances existed in two forms. We observed double signals of the alpha proton of the amino acid residue, and protons of the nearest environment. This phenomenon did not depend on the amino acid chirality, and was observed in both L- and D-amino acids as well as in optically inactive glycine, beta-alanine and ε-lysine. The difference in the chemical shifts of the alpha-proton signals of the amino acid residue was significant (more than 0.8 ppm). 

We decided to investigate whether the mini-form can be formed due to the appearance of a hindered structure with an intramolecular hydrogen bond. Our aim was to determine the localization of this hydrogen bond, and confirm our theory with two-dimensional heteronuclear NMR spectroscopy data.

## 2. Results

**Figure 4:**
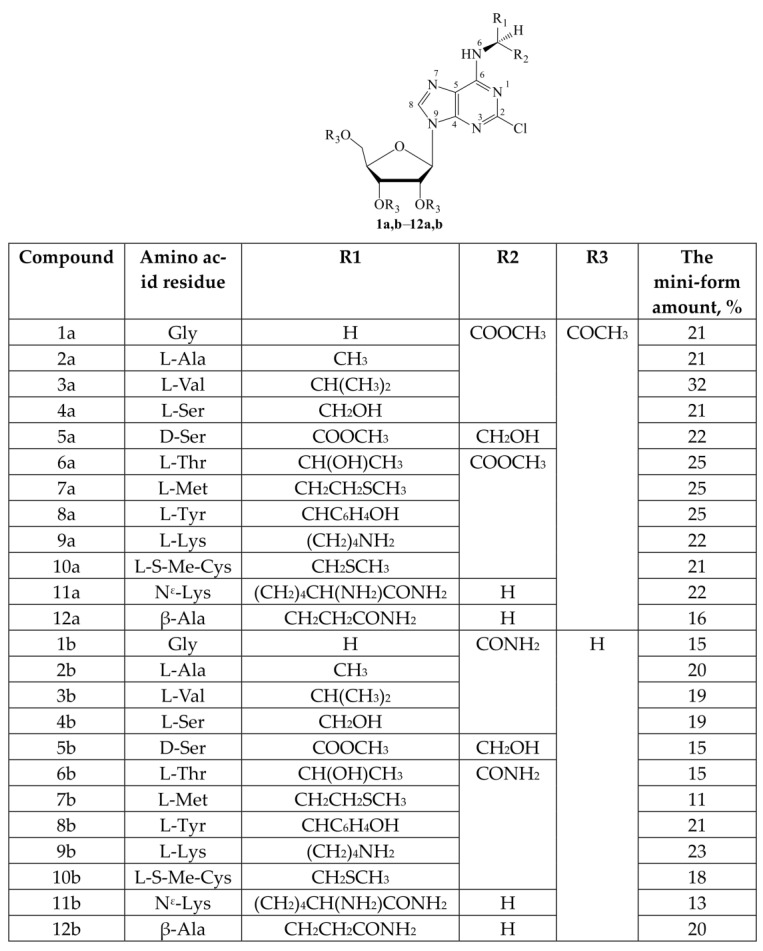
The amount of mini-form in nucleoside NMR spectra.

The NMR spectra analysis of acylated nucleosides **1a**–**12a** and ribosides **1b**–**12b** [5] revealed the presence of a significant amount of the nucleoside second form. The content of the mini-form component for nucleosides **1a**,**b**–**12a**,**b** varied from 11 to 32% of the main form (Figure 4). According to HPLC and LC-MS, all products were homogeneous.

The signal of the N^6^CH proton was significantly shifted downfield (see Table 1), as well as N^6^CH carbon and N^6^H nitrogen signals. Heavy atoms of the purine heterocycle were also shifted downfield (N7, C4) or upfield (C8, C5, C2). The chemical shifts of the amide group protons were shifted upfield. They are not shown in the table, because it is difficult to calculate the difference in chemical shifts between the two forms of the dual set of signals. The exception is the N^6^H proton. It can be shifted both upfield and downfield.

**Figure 5:**
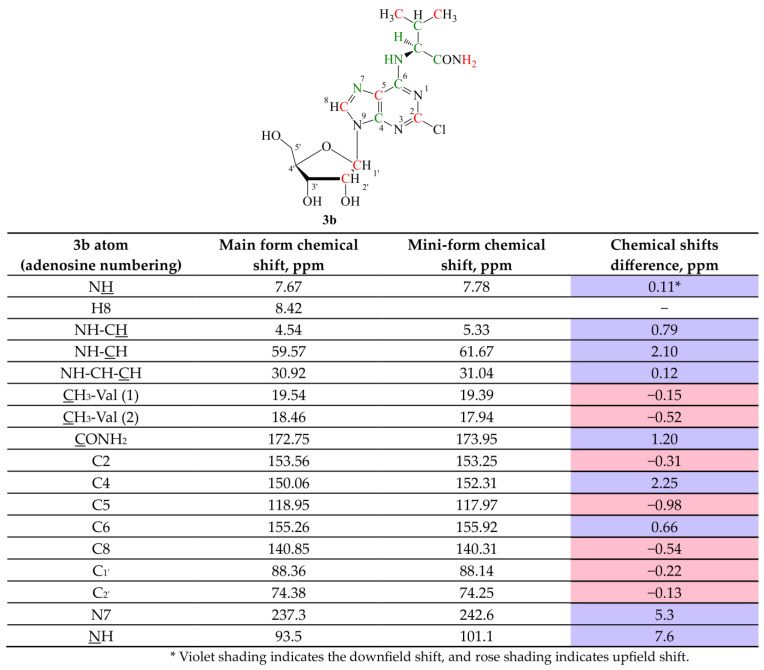
The differences in the chemical shifts for mini-form and main form atoms of nucleoside **3b**.

The NMR spectra set of valine derivative **3b** was chosen to represent changes in chemical shifts because of the considerable amount of mini-form in the NMR spectra (See Figures S5–S10 in the Supplementary Materials). The 1D and 2D NMR spectra were recorded again after preparing the sample with an increased concentration of valine derivative **3b** (770 mg/mL) to improve the signal-to-noise value. The mini-form had the same set of signals as the main form in COSY, ^1^H,^13^C-HMBC and ^1^H,^15^N-HMBC spectra (See Figures S11–S16 in the Supplementary Materials). Differences in the chemical shifts of main and mini-form atoms are shown in Figure 5.

**Figure 6:**
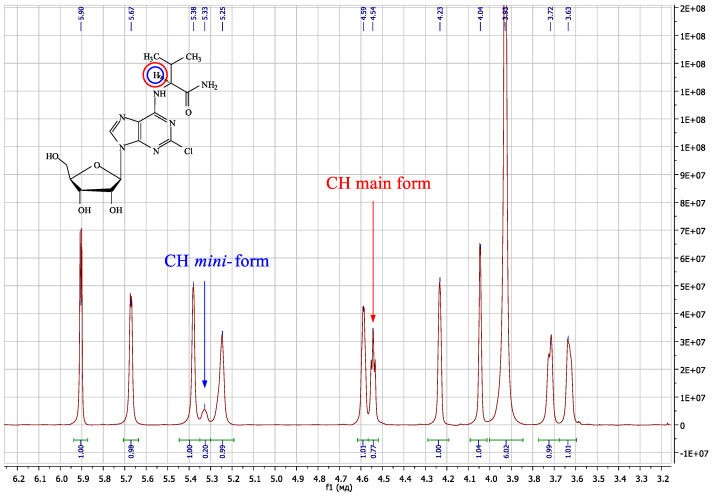
Fragment of valine derivative **3b** ^1^H NMR spectrum. The N^6^CH proton chemical shifts are shown.

The chemical shift of the ^α^CH proton of the valine residue changed by 0.79 ppm according to the NMR ^1^H spectrum (Figure 6). The chemical shift of the CH proton of the main form **3b** was 4.54 ppm, while the signal of the mini-form was shifted downfield to 5.33 ppm. The further the protons were from the chiral center of the amino acid, the less this effect was pronounced (Figure 5).

**Figure 7:**
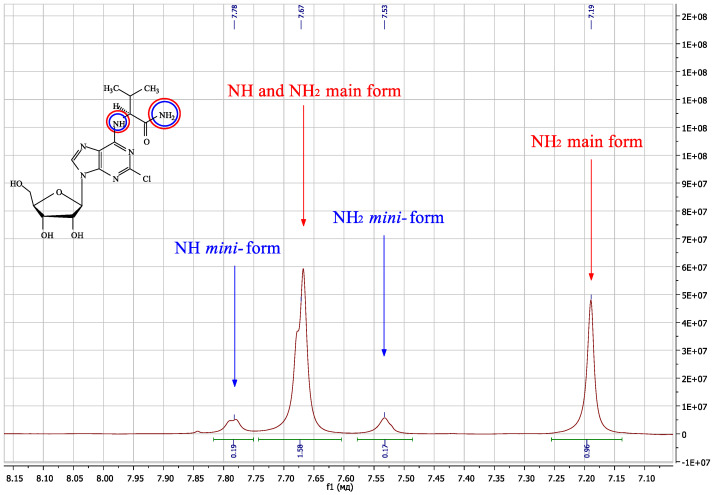
Fragment of valine derivative **3b** ^1^H NMR spectrum. The NH and NH_2_ groups protons chemical shifts are shown.

**Figure 8:**
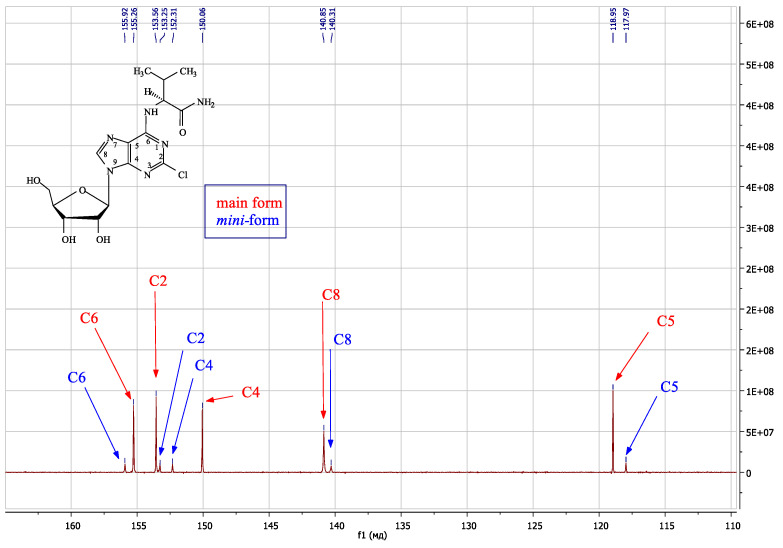
Fragment of valine derivative **3b** ^13^C NMR spectrum.

**Figure 9:**
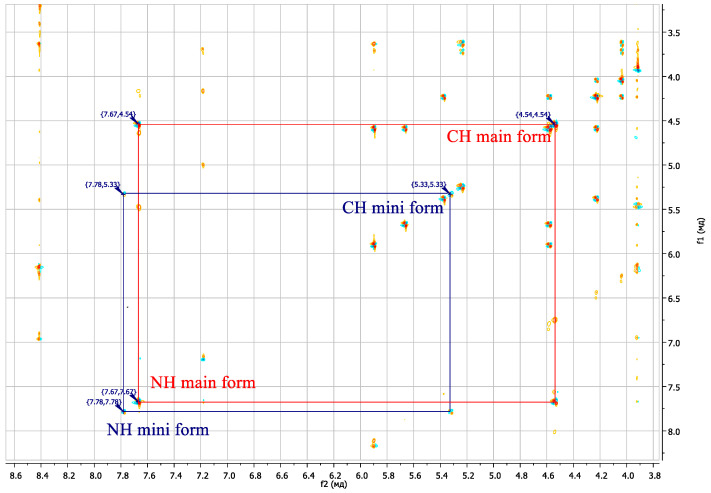
Fragment of valine derivative **3b** ^1^H COSY spectrum. Red lines are for the main form, blue lines are for the mini form.

The dual set of signals is also presented for the amine and amide group protons (Figure 7). The separate set of signals from the nucleoside mini-form was also registered in ^13^C NMR spectra (see Figure 8) and COSY spectra (Figure 9).

**Figure 10:**
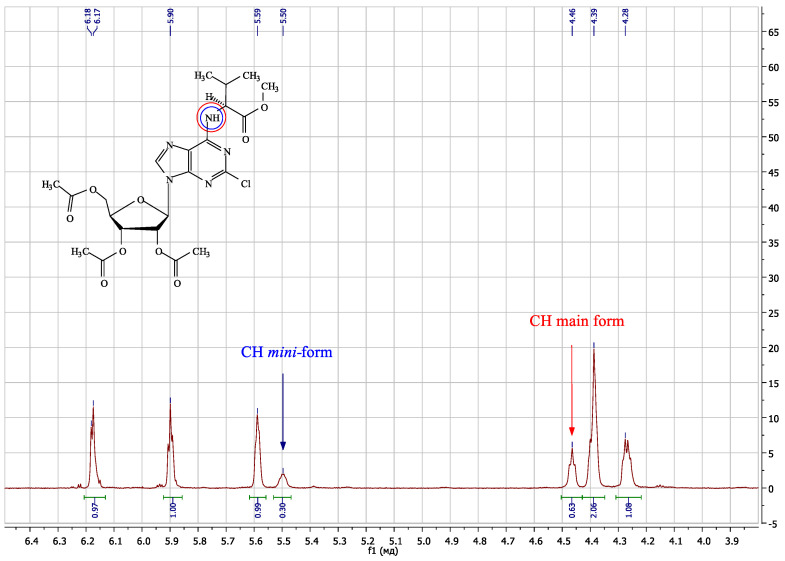
Fragment of valine derivative triacetate **3a** ^1^H NMR spectrum.

The mini-form was observed not only for ribosides **1b**–**12b**, but also for protected nucleosides **1a**–**12a** (Figure 10, a fragment of the ^1^H NMR spectrum of valine methyl ester triacetate **3a** is shown; see Figures S1–S4 in the Supplementary Materials). The appearance of the nucleoside mini-form did not require an amide group or a methyl ester.

**Figure 11:**
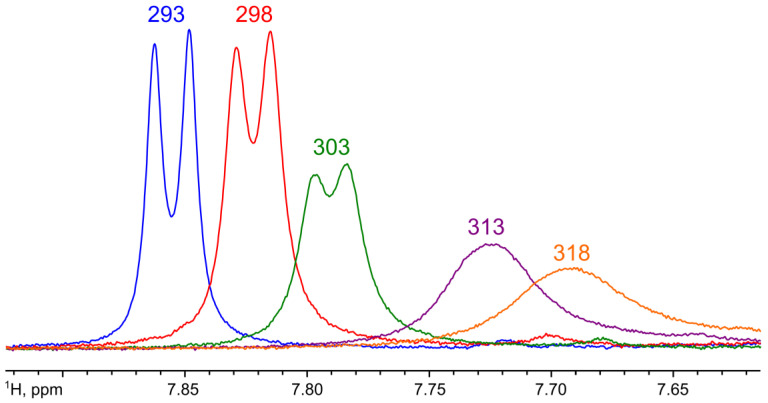
Temperature dependence of the shape and chemical shift of N^6^H signal in **3b** mini-form ^1^H NMR. Temperatures are given in Kelvin.

We studied the temperature effect on the mini-form formation (Figure 11). With a temperature increase from 293 to 318 K, the signals of the same protons of the main and mini-form began to broaden. This broadening is evidence of a chemical exchange process well known in NMR spectroscopy [31]. This NMR evidence of the chemical exchange provided an independent proof that we have two forms of the same substance; the mini-form interchanged with the main form during the NMR experiment. The ^1^H signals of the main form and the mini-form did not merge into each other, but that would occur at higher temperatures not available on our NMR equipment. In our experiments at 293–318 K, the content of the second form in NMR did not change.

**Figure 12:**
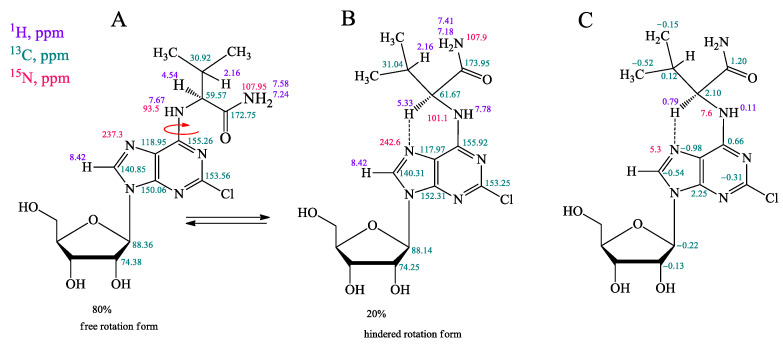
Chemical shifts of the main component (**A**) and the mini-form with N^6^CH∙∙∙N7 hydrogen bond (**B**) of **3b**, and differences between them (**C**). Only the chemical shifts that differ between the forms are shown. Full assignment is given in the Supplementary Materials (Figure S34).

The N^6^H proton of each form had a cross-peak with the N^6^CH proton in the COSY NMR spectrum. This discards the amino–imino tautomerism. We suggest that hindered rotation appeared. In the main form, the amino acid substituent rotated freely around the C6–N^6^ bond, while in the mini-form the structure was in a hindered state (Figure 12). We assumed that the hindered structure was caused by the intramolecular hydrogen bond formation. The hydrogen bond was formed between the N^6^CH proton and the N7 purine atom, with a stable six-membered ring formed. The proposed hindered structure is shown in Figure 12.

**Figure 13:**
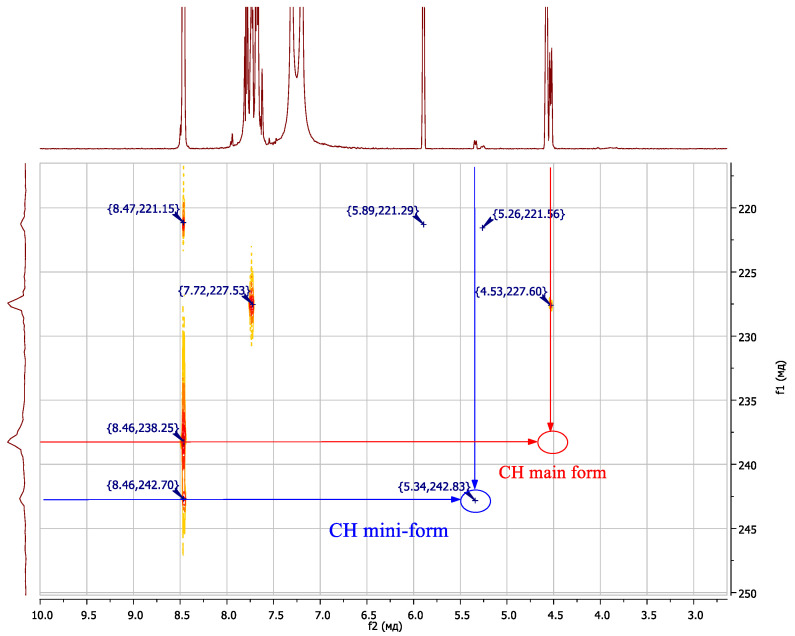
The fragment of ^1^H,^15^N-HMBC spectrum of the valine derivative **3b** (293 K).

The presence of a hydrogen bond can be detected by the NMR spectrum, both for purine heterocycle [32] and the amino acid [33]. This should result in a cross peak between the N^6^CH proton and the purine N7 in the ^1^H,^15^N-HMBC spectrum. This peak was observed in the spectrum of the **3b** mini-form, with no peak for the main form (Figure 13). This proves the presence of the hydrogen bond. 

**Figure 14:**
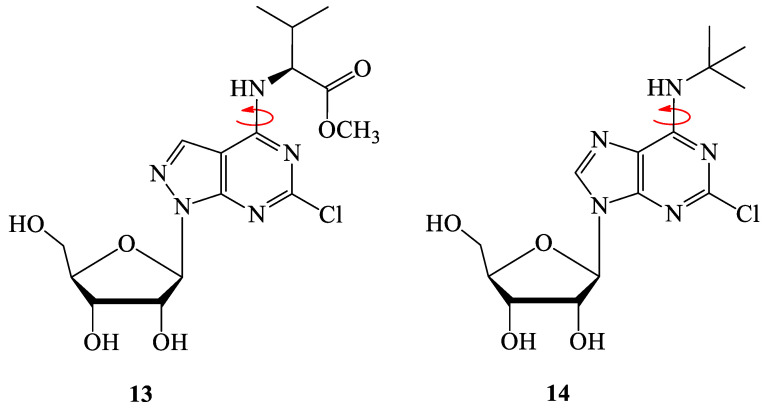
Structures of nucleosides incapable of forming the N^6^CH∙∙∙N7 intramolecular hydrogen bond. Red arrows show free rotation of C6 substituent.

It was necessary to prove the presence of this hydrogen bond to finally confirm our theory. Several new nucleosides that have been synthesized are incapable of forming N^6^CH∙∙∙N7 hydrogen bonding, either because of the absence of the nitrogen atom in the N7 position (8-aza-7-deaza-adenosine derivative **13**) or because of the N^6^CH proton’s absence (tert-butylamino derivative **14**) (Figure 14).

**Scheme 1:**
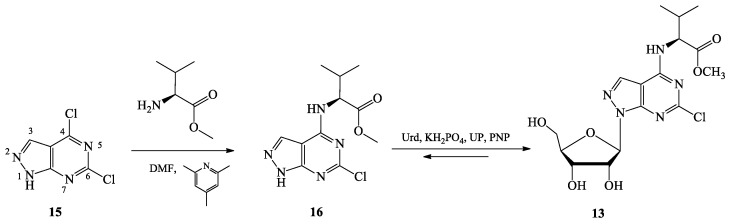
Synthesis of 8-aza-7-deazaadenosine derivative **13**.

The synthesis of 8-aza-7-deaza-adenosine derivative **13** was carried out according to Scheme 1. The starting 2,6-dichloroallopurinol **15** reacted with L-valine methyl ester in the presence of 2,4,6-collidine. Heterocycle **16** was ribosylated by a transglycosylation reaction with uridine phosphorylase (UP) and purine nucleoside phosphorylase (PNP) [34] to give product **13**.

**Scheme 2:**
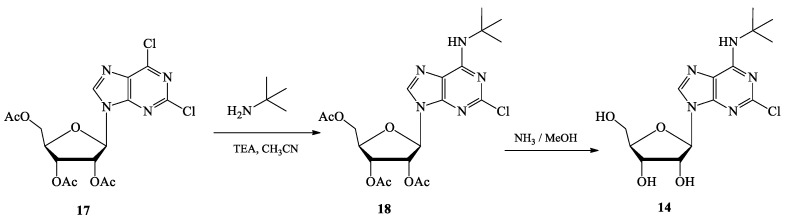
Synthesis of tert-butylamine derivative **14**.

The synthesis of tert-butylamino derivative **14** was carried out according to Scheme 2. The starting 2,6-dichloro-9-(2′,3′,5′-tri-O-acetyl-*β*-*D*-ribofuranosyl)purine **17** was obtained according to the procedure published earlier [5]. The reaction was carried out in acetonitrile in the presence of triethylamine. To remove the acetyl protection groups from the ribose, product **18** was treated with a saturated solution of ammonia in methanol at 4 °C to obtain the corresponding nucleoside **14**.

The nucleosides **13** and **14** were characterized by ^1^H, ^13^C, COSY, ^1^H,^13^C HSQC, ^1^H,^13^C HMBC, ^1^H,^15^N HSQC and ^1^H,^15^N HMBC NMR spectra. NMR spectra are provided in the Supplementary Materials (Figures S17–S33). No second form of nucleosides was observed in NMR (Figure 15 and Figure 16).

The theory of the appearance of the mini-form by amino–imino tautomerism turned out to be untenable for our compounds. We have explained the appearance of the second form in the NMR spectra of N^6^-substituted 2-chloroadenosine nucleosides by the formation of an intramolecular hydrogen bond between the proton of the N^6^CH substituent and the N7 atom of the purine base. The content of the mini-form was independent of temperature. The mini-form was not observed in the NMR spectra of compounds lacking either the purine N7 atom or the N^6^CH proton of the substituent.

## 3. Discussion

There are several hypotheses explaining the presence of the mini-form in the NMR spectra of purine nucleosides. The first is that the appearance of a second form in the NMR spectra of 6-substituted adenosines is attributed to amino–imino tautomerism [12] (tautomerism hypothesis). For the second, the mini-form is attributed to the hindered rotation [11,13,14,15]. We propose here that the hindered structure is stabilized by the non-conventional intramolecular CH∙∙∙N7 hydrogen bond formation (H-bond hypothesis) (Figure 17).

NMR chemical shifts exhibit high sensitivity to any alterations in the chemical or spatial structure of a molecule. Our initial assumption was that analyzing the NMR data would uncover consistent patterns in the chemical shift variations of the atoms implicated in the hindered structure formation. Unfortunately, the chemical shifts presented in Table 1 do not provide definitive conclusions, making it challenging to favor one hypothesis over another. 

In our research involving various 2-chloro-C6-substituted purine ribonucleosides [5], we observed cross-peaks between the N^6^H and CH proton signals in the COSY spectrum in both main and mini-form in all compounds considered (Figure 9, for compound **3b**), leading us to doubt the tautomerism hypothesis. On the other hand, the H-bond hypothesis assumes COSY cross-peak presence in both main and mini forms, as seen in our experiments. 

We could keep the tautomerism hypothesis in consideration by examining the possibility of an amide-like C6–N^6^ partial double bond instead of an imino double bond. However, we found this explanation to be unsuitable. Despite the fact that it would account for the stable planar hindered structure, the chemical shift of the N^6^H proton did not exhibit significant changes (Table 1). Notably, the N^6^H proton exhibited both upfield and downfield shifts across different compounds. 

Moreover, amino–imino tautomerism should drastically influence the ^15^N chemical shift of the N^6^H nitrogen. We would expect significant chemical shift changes of around one hundred ppm on the basis of available experimental data and quantum-theory calculations [35,36]. However, contrary to these expectations, our experimental data revealed only 4–20 ppm changes in the N^6^H nitrogen chemical shift between the main and mini forms (Table 1). Hence, we must reject the amino–imino tautomerism hypothesis that was previously proposed [12], as it is not consistent with the experimental data obtained in the present work.

To further explore the H-bond hypothesis, we obtained the ^15^N-HMBC spectrum at an increased concentration of the substance **3b** (770 mg/mL in DMSO-*d*_6_). This spectrum revealed a cross-peak between the purine N7 atom and the N^6^CH proton of mini-form (Figure 13), indicating the presence of long-range through-bond interactions (Figure 17). Notably, this cross-peak was not observed in the main form. In the case of the tautomerism hypothesis this cross peak could be explained as five bonds correlation only (Figure 17, bold lines), and we could not understand its absence in the main form. Really, it would be predominantly observed in the main form, which constitutes approximately 80% of the total. Hence, that cross-peak makes the H-bond hypothesis highly preferable in comparison with the tautomerism hypothesis. 

In general, cross-peaks in multidimensional NMR spectra appear either because of spin–spin coupling between two nuclei (through-bond connectivities) or because of through-space cross-relaxation effects (NOESY or ROESY interactions). The ^1^H,^15^N-HMBC NMR experiment used in the present work revealed cross-peaks that appeared because of multiple-bond long range spin–spin couplings from ^1^H to ^15^N nuclei. Similar long-range couplings have been exploited in especially designed NMR techniques used for hydrogen bond detection in proteins [33] and nucleic acid base pairs [32]. Moreover, exactly the same ^1^H,^15^N-HMBC experiment has been employed to confirm intramolecular O–H···N [37] or N–H···N [38] hydrogen bonding. Several reviews have extensively discussed numerous examples of different hydrogen bonds verified by NMR [39,40]. However, to the best of our knowledge, the utilization of the ^1^H,^15^N-HMBC NMR experiment for the detection of non-conventional CH∙∙∙N7 bonds has not been previously reported.

Additionally, we observed a distinct hydrogen bond between 5′-OH and purine N3 [41] in the spectrum (Figure 13, cross-peak {5.26, 221.56}). This spatial proximity and the presence of a hydrogen bond suggest that such bond types are detectable on this 2D HMBC spectrum when the substance is at an elevated concentration.

**Figure 15:**
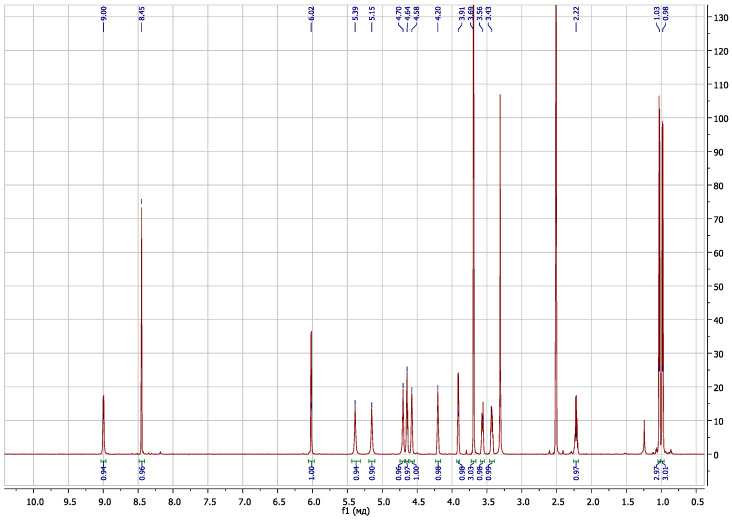
The allopurinol nucleoside **13** ^1^H NMR spectrum (303 K).

**Figure 16:**
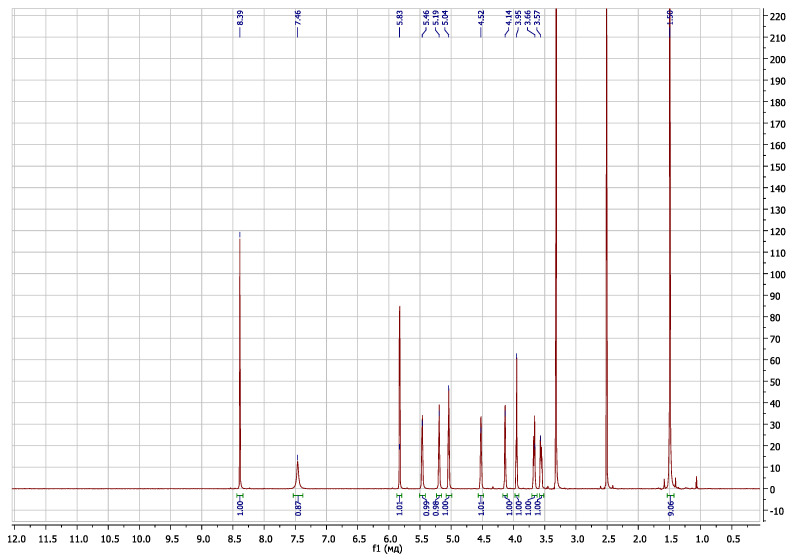
The nucleoside **14** ^1^H NMR spectrum (303 K).

**Figure 17:**
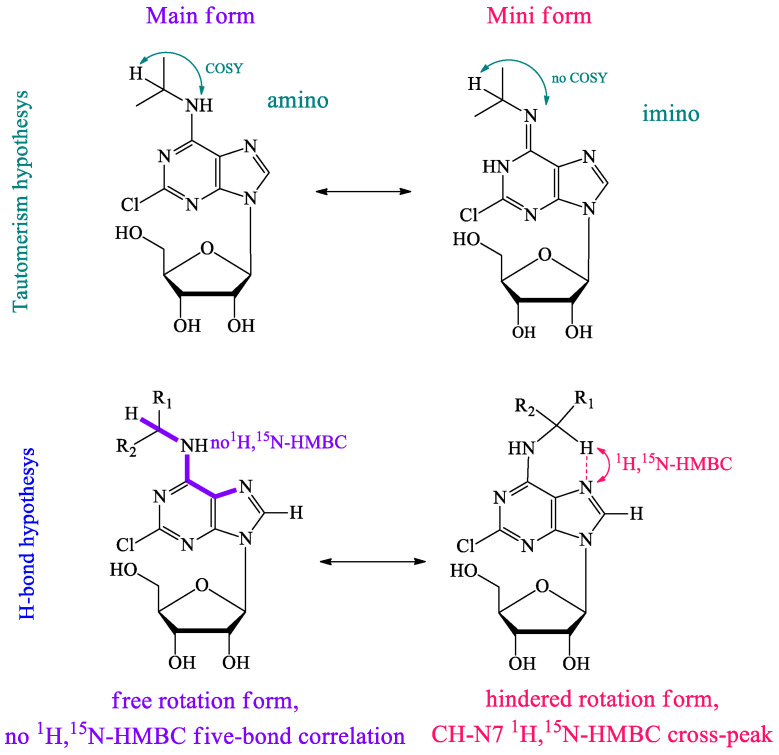
Two hypotheses of the occurrence of the mini-form.

The presence of the mini-form in various structures was found irrespective of substituent chirality, including L- and D-amino acids (**4b** and **5b**), non-chiral compounds (**11b** and **12b**) and bulky substituent (**8b**), and suggests its inherent nature. In the absence of the N^6^CH proton, the formation of a mini-form does not occur (compound **14**, Figure 16), underscoring its critical role in the occurrence of hindered rotation. We observed significant downfield chemical shift changes, predominantly for the N^6^CH proton, providing further evidence of its involvement in the formation of the hindered rotation conformation. Furthermore, the absence of the N7 purine atom in compound **13** corresponds to the absence of the mini-form (Figure 15), further supporting the H-bond hypothesis and rejecting the tautomerism hypothesis. 

The formation of the mini-form can be attributed to restricted rotation, which arises not from steric factors but rather from the presence of a stabilizing hydrogen bond. The main form appears to represent a position of the N^6^-substituent, where no hydrogen bond is formed. Future investigations will delve into the study of the intramolecular hydrogen bond formation in different solvents and under cooling conditions.

In conclusion, the full set of experimental data presented provides unambiguous evidence for the presence of a non-conventional CH∙∙∙N hydrogen bond in 2-chloro-C6-substituted purine ribosides. Alternative explanations involving amino–imino tautomerism have been ruled out due to their inconsistency with the NMR data obtained in this study.

## 4. Materials and Methods

All solvents and chemicals were used as purchased without further purification. Valine derivative **3b** was synthesized according to a previously developed procedure [5]. The progress of reactions was monitored on Silufol precoated silica gel plates (with fluorescence indicator UV_254_) using methanol/chloroform as a solvent system. Spots were visualized through irradiation with ultraviolet light (254 nm). Analytical high-performance liquid chromatography (HPLC) was performed on a Waters system (Waters 1525, Waters 2489, Breeze 2) using Nova Pak C18 column, 4.6 × 150 mm, 5 µm, flow rate 1 mL/min, eluent A—H_2_O/0.1% TFA, eluent B—70% CH_3_CN/H_2_O/0.1% TFA, gradient: 0–100% B over 20 min), detection at 254 nm. Column chromatography was performed on Silica gel 60 or C18-reversed-phase silica gel (Fluka, Buchs, Switzerland).

NMR spectra were acquired on a Bruker Avance I 700 MHz or Bruker Avance III 800 MHz NMR in DMSO-*d*_6_ at 303 K. To study the temperature dependence, the spectra were taken at 293, 298, 303, 313 and 318 K. Chemical shifts in ppm (δ) were measured relative to the residual solvent signals as internal standards (2.50). Coupling constants (J) were measured in Hz. Liquid chromatography mass spectrometry was performed using an Agilent 6210 TOF LC–MS system (Agilent Technologies, Santa Clara, CA, USA). 

**9-*β*-*D*-ribofuranosyl-2-chloro-6-(N^α^-L-valinylamido)-purine (3b)**

Nucleoside **3b** was synthesized according to a previously developed procedure [5].

**Main form**: ^1^H NMR (800 MHz, DMSO-*d*_6_, *J*, Hz, 303 K): δ 8.42 (s, 1 H, H 8), 7.67 (m, 0.81 H, NH), 7.67 and 7.19 (2 s, 0.77 and 0.91 H, NH_2_), 5.90 (d, *J* = 5.8 Hz, 1 H, H_1′_), 5.67 (d, *J* = 6.2 Hz, 1 H, OH_2′_), 5.38 (s, 1 H, OH_3′_), 5.25 (m, 1 H, OH_5′_), 4.59 (m, 1 H, H_2′_), 4.54 (m, 0.77 H, ^α^CH-Val), 4.23 (m, 1 H, H_3′_), 4.04 (m, 1 H, H_4′_), 3.72 (m, 1 H, H_5′a_), 3.63 (m, 1 H, H_5′b_), 2.16 (m, 1 H, ^β^CH-Val), 0.90 (m, 6 H, CH_3_-Val). ^13^C NMR (176 MHz, DMSO-*d*_6_, 303 K): δ 172.75 (CONH_2_), 155.26 (C6), 153.56 (C2), 150.06 (C4), 140.85 (C8), 118.95 (C5), 88.36 (C_1′_), 86.25 (C_4′_), 74.38 (C_2′_), 70.92 (C_3′_), 61.85 (C_5′_), 59.57 (^α^CH-Val), 30.92 (^β^CH-Val), 19.54 (CH_3_-Val), 18.46 ppm (CH_3_-Val). ^15^N NMR (71 MHz, DMSO-*d*_6_, 303 K): δ 237.28 (N7), 227.62 (N1), 221.20 (N3) 171.28 (N9), 107.95 (NH_2_), 93.53 ppm (NH).

**Mini**-**form:** ^1^H NMR (800 MHz, DMSO-*d*_6_, *J*, Hz, 303 K): δ 8.42 (s, 1 H, H 8), 7.78 (m, 0.19 H, NH), 7.53 (s, 0.17 H, NH_2_), 5.90 (d, *J* = 5.8 Hz, 1 H, H_1′_), 5.67 (d, *J* = 6.2 Hz, 1 H, OH_2′_), 5.38 (s, 1 H, OH_3′_), 5.33 (m, 0.20 H, ^α^CH-Val), 5.25 (m, 1 H, OH_5′_), 4.59 (m, 1 H, H_2′_), 4.23 (m, 1 H, H_3′_), 4.04 (m, 1 H, H_4′_), 3.72 (m, 1 H, H_5′a_), 3.63 (m, 1 H, H_5′b_), 2.16 (m, 1 H, ^β^CH-Val), 0.90 (m, 6 H, CH_3_-Val). ^13^C NMR (176 MHz, DMSO-*d*_6_, 303 K): δ 173.95 (CONH_2_), 155.92 (C6) 153.25 (C2), 152.31 (C4), 140.31 (C8), 117.97 (C5), 88.14 (C_1′_), 74.25 (C_2′_), 61.67 (^α^CH-Val), 31.04 (^β^CH-Val), 19.39 (CH_3_-Val), 17.94 ppm (CH_3_-Val). ^15^N NMR (71 MHz, DMSO-*d*_6_, 303 K): δ 242.57 (N7), 227.62 (N1), 221.20 (N3) 171.28 (N9), 107.92 (NH_2_), 101.12 ppm (NH).

**6-chloro-4-(N^α^-L-valinyl)-pyrazolo[3,4-d]pyrimidine methyl ester (16)**

To a solution of 150.0 mg (0.794 mmol) of 4,6-dichloro-1H-pyrazolo[3,4-d] and 234.0 mg (1.40 mmol) of L-valine methyl ester hydrochloride in 3 mL of dry DMF was added 300 µL (275.1 mg, 2.273 mmol) of 2,4,6-collidine at room temperature. The mixture was incubated at 50 °C for 24 h. The reaction progress was monitored by HPLC. The solvent was removed in vacuo, and the residue was dissolved in a minimal volume of water. The desired product was isolated by flash column chromatography on C18 silica gel (10 × 1.5 cm, elution by EtOH in H_2_O, 10%). Then, 87.9 mg (39.0%) of the product was obtained as a white solid with a purity of 97.06%. ^1^H NMR (700 MHz, DMSO-*d*_6_, *J*, Hz, 303 K): δ 13.60 (s, 0.89 H, N1–H), 8.81 (d, *J* = 7.4 Hz, 1 H, NH-Val), 8.35 (s, 1 H, H3), 4.62 (m, 1 H, ^α^CH-Val), 3.69 (s, 3 H, −OCH_3_), 2.21 (m, 1 H, ^β^CH-Val), 1.02 (d, *J* = 6.2 Hz, 3 H, CH_3_), 0.98 ppm (d, *J* = 6.3 Hz, 3 H, CH_3_). ^13^C NMR (176 MHz, DMSO-*d*_6_, 303 K): δ 171.73 (COOCH_3_), 156.58 (C4), 155.35 (C6 or C7a), 133.14 (C3), 98.65 (C4a), 58.87 (^α^CH-Val), 51.70 (–OCH_3_), 29.99 (^β^CH-Val), 18.81 (CH_3_), 18.74 ppm (CH_3_). ^15^N NMR (71 MHz, DMSO-*d*_6_, 303 K): δ 221.5 (N5), 190.8 (N1), 101.9 ppm (NH-Val). HRMS (ESI) C_11_H_14_N_5_O_2_Cl [M − H]^−^: 282.0758 calcd., 282.0768 found.

**6-chloro-1-*β*-*D*-ribofuranosyl-4-(N^α^-L-valinyl)-pyrazolo[3,4-d]pyrimidine methyl ester (13)**

To a solution of 41 mg of uridine (0.168 mmol), 30 mg of 6-chloro-4-(N^α^-L-valinyl)-pyrazolo[3,4-d]pyrimidine methyl ester **16** (0.106 mmol), and 148 mg of KH_2_PO_4_ (1.109 mmol) in 212 mL of distilled water was added 3.0 µL (4.2 units) of *E. coli* PNP and 2.4 µL (4.1 units) of *E.coli* UP. The pH was adjusted up to 7.0 with 5 M KOH solution and the reaction mixture was incubated at 50 °C. The reaction progress was monitored with HPLC. When conversion reached the highest value, the reaction was terminated by the addition of ethanol (40%, *v*/*v*). The reaction mixture was evaporated up to 5 mL, and the desired product was isolated using reversed-phase column chromatography (silica gel C18, Merck), column 2.5×3.0 cm (elution by EtOH in H_2_O, 40%). Then, 30.4 mg (69.0%) of the product was obtained as a white solid with a purity of 97.86%. ^1^H NMR (700 MHz, DMSO-*d*_6_, *J*, Hz, 303 K): δ 9.00 (d, *J* = 7.2 Hz, 1 H, NH-Val), 8.45 (s, 1 H, H3), 6.02 (d, *J* = 4.6 Hz, 1 H, H_1′_), 5.39 (m, 1 H, OH_2′_), 5.15 (m, 1 H, OH_3′_), 4.70 (m, 1 H, OH_5′_), 4.64 (m, 1 H, ^α^CH-Val), 4.58 (m, 1 H, H_2′_), 4.20 (m, 1 H, H_3′_), 3.91 (m, 1 H, H_4′_), 3.69 (s, 3 H, OCH_3_), 3.56 (m, 1 H, H_5′a_), 3.43 (m, 1 H, H_5′b_), 2.22 (m, 1 H, ^β^CH-Val), 1.03 (d, *J* = 6.6 Hz, 3 H, CH_3_), 0.98 ppm (d, *J* = 6.8 Hz, 3 H, CH_3_). ^13^C NMR (176 MHz, DMSO-*d*_6_, 303 K): δ 171.53 (COOCH_3_), 157.03 (C6), 156.60 (C4), 154.72 (C7a), 133.85 (C3), 99.55 (C4a), 88.10 (C_1′_), 85.15 (C_4′_), 72.95 (C_2′_), 70.68 (C_3′_), 62.13 (C_5′_), 58.96 (^α^CH-Val), 51.78 (OCH_3_), 30.01 (^β^CH-Val), 18.79 (CH_3_), 18.68 ppm (CH_3_). ^15^N NMR (71 MHz, DMSO-*d*_6_, 303 K): δ 305.3 (N2), 222.8 (N5), 199.5 (N1), 102.5 ppm (NH-Val). HRMS (ESI) C_16_H_22_N_5_O_6_Cl [M − H]^−^: 414.1180 calcd., 414.1194 found.

**9-(2′,3′,5′-Tri-*O*-acetyl-*β*-*D*-ribofuranosyl)-2-chloro-6-*tert*-butylamino-purine (18)**

To a solution of 30.0 mg (0.067 mmol) of 2,6-dichloro-9-(2**′**,3**′**,5**′**-tri-*O*-acetyl-*β*-*D*-ribofuranosyl)purine and 20 µL (14 mg, 0.188 mmol) of tert-butylamine (Sigma-Aldrich) in 1 mL of dry DMF was added 50 µL (36 mg, 0.360 mmol of triethylamine. The mixture was stirred at room temperature for 18 h, and the progress was monitored with thin-layer chromatography (TLC) on silica gel (chloroform–methanol, 30:1). The solvent was removed in vacuo, and the residue was dissolved in a minimal volume of chloroform, and the desired product was isolated using column chromatography on silica gel (10 × 1.5 cm, with elution with MeOH gradient in CHCl_3_, 0–20%). Then, 32.1 mg (98.7%) of the product was obtained as a yellow solid with a purity of 98.47%. ^1^H NMR (700 MHz, DMSO-*d*_6_, *J*, Hz, 303 K): δ 8.39 (s, 1 H, H8), 7.57 (br.s, 0.76 H, NH), 6.15 (d, *J* = 5.5, 1 H, H_1′_),5.90 (t, *J* = 5.7 Hz, 1 H, H_2′_), 5.58 (m, 1 H, H_3′_), 4.39 (m, 2 H, H_5′a_ and H_4′_), 4.27 (m, 1 H, H_5′b_), 2.13 (m, 3 H, CH_3_CO-3′), 2.05 (m, 3 H, CH_3_CO-2′), 2.03 (m, 3 H, CH_3_CO-5′), 1.49 ppm (s, 9 H, CH_3_). ^13^C NMR (176 MHz, DMSO-*d*_6_, 303 K): δ 169.88 (O–CO^5′^), 169.29 (O–CO^3′^), 169.13 (O–CO^2′^), 154.68 (C6)_,_ 152.43 (C2)_,_ 139.72 (C8), 85.27 (C_1′_), 79.47 (C_4′_), 71.97 (C_2′_), 69.86 (C_3′_), 62.60 (C_5′_), 52.08 (C(CH_3_)_3_), 28.46 (C(CH_3_)_3_), 20.35 (CH_3_–CO^5′^), 20.24 (CH_3_–CO^3′^), 20.08 ppm (CH_3_–CO^2′^). ^15^N NMR (71 MHz, DMSO-*d*_6_, 303 K): δ 243.0 (N7), 167.3 (N9), 114.2 ppm (NH). HRMS (ESI) C_20_H_26_N_5_O_7_Cl [M − H]^−^: 482.1442 calcd., 482.1438 found.

**9-*β*-*D*-Ribofuranosyl-2-chloro-6-*tert*-butylamino-purine (14)**

A total of 24 mg (0.066 mmol) of nucleoside triacetate **18** was dissolved in 5 mL of absolute methanol and cooled in an ice bath. The solution was saturated with ammonia and kept for 24 h at +4 °C. The reaction progress was controlled by TLC on silica gel (chloroform–methanol 10:1). After reaction completion, the solvent was evaporated, the residue was dissolved in a minimal volume of water, and the desired product was isolated with column chromatography on C_18_-reversed-phase silica gel (12 × 2 cm, elution with EtOH gradient in water, 10–80%). Then, 15.3 mg (86.5%) of the product was obtained as a yellow solid with a purity of 98.80%. ^1^H NMR (700 MHz, DMSO-*d*_6_, *J*, Hz, 303 K): δ 8.39 (s, 1 H, H8), 7.46 (br.s, 0.87 H, NH), 5.83 (d, *J* = 5.9 Hz, 1 H, H_1′_), 5.46 (m, 1 H, OH_1′_), 5.19 (m, 1 H, OH_2′_), 5.04 (t, *J* = 5.6 Hz, 1 H, OH_5′_),4.52 (m, 1 H, H_2′_), 4.14 (m, 1 H, H_3′_), 3.95 (m, 1 H, H_4′_), 3.66 (m, 1 H, H_5′a_), 3.57 (m, 1 H, H_5′b_), 1.50 ppm (s, 9 H, CH_3_).^13^C NMR (176 MHz, DMSO-*d*_6_, 303 K): δ 154.67 (C6)_,_ 152.16 (C2)_,_ 149.23 (C4), 139.54 (C8), 118.86 (C5), 87.31 (C_1′_), 85.60 (C_4′_), 73.55 (C_2′_), 70.25 (C_3′_), 61.26 (C_5′_), 52.02 (NHCH), 28.52 ppm (CH_3_). ^15^N NMR (71 MHz, DMSO-*d*_6_, 303 K): δ 241.1 (N7), 218.9 (N3), 170.9 (N9), 113.4 ppm (NH). HRMS (ESI) C_14_H_20_N_5_O_4_Cl [M − H]^−^: 356.1125 calcd., 356.1120 found.

## 5. Conclusions

During the examination of the NMR spectra of N^6^-substituted 2-chloroadenosines, a second form was discovered. This form, present at 11–32% of the main form, was identified by a unique set of signals in the 2D NMR spectra. The mini-form’s temperature dependence was investigated and found to remain constant in quantity, but the peak shape and chemical shift were affected. Our hypothesis proposes that the presence of the mini-form is due to restricted rotation caused by the formation of an intramolecular hydrogen bond between the N7 atom of purine and the N^6^CH proton of the substituent. This was confirmed by the key cross-peak in the ^1^H,^15^N-HMBC NMR spectrum from the CH donor to the N7 acceptor presented in the mini-form but not in the main form. We synthesized compounds that lacked either the donor (CH) or acceptor (N7) of the proposed N^6^CH∙∙∙N7 hydrogen bond. The mini-form was absent in the NMR spectra of these compounds, supporting the importance of the intramolecular hydrogen bond in its formation. The full set of experimental NMR data has provided unambiguous evidence for the presence of a non-conventional CH∙∙∙N hydrogen bond in 2-chloro-C6-substituted purine ribosides. This type of hydrogen bond was discovered using ^1^H,^15^N-HMBC NMR spectrum in a purine nucleoside in a first time.

## Figures and Tables

**Table 1:** The chemical shifts difference between the mini-form and the main form. Shading has been introduced for clarity. Violet background color indicates the downfield shift, and rose indicates an upfield shift.

Compound	Amino Acid Residue	Δδ N^6^CH, ppm	Δδ N^6^H, ppm	Δδ C8H, ppm	Δδ N^6^CH, ppm	Δδ N^6^H, ppm	Δδ N7, ppm	Δδ C4, ppm
**1a**	Gly	0.42	−0.20	−0.06	2.46	− *	−	−
**2a**	L-Ala	0.72	−0.17	−0.07	2.4	6.6	−	−
**3a**	L-Val	1.04	−0.13	−0.07	1.22	4.1	−	−
**4a**	L-Ser	0.73	−0.05	−0.07	2.57	7.9	1.4	1.91
**5a**	D-Ser	0.73	−0.05	−0.08	2.6	7.5	1.2	1.88
**6a**	L-Thr	0.79	0.03	−0.09	2.45	6.9	−	1.97
**7a**	L-Met	0.72	−0.19	−0.07	2.13	6.4	−	−
**8a**	L-Tyr	0.83	−0.10	−0.06	2.96	6.6	1.4	1.91
**9a**	L-Lys	0.75	−0.16	−0.07	2.43	7.6	−	1.80
**10a**	L-S-Me-Cys	0.86	−0.14	−0.04	2.93	7.1	−	1.83
**11a**	N^ε^-Lys	0.40	−0.04	−0.08	2.14	−	−	−
**12a**	β-Ala	0.40	−0.04	−0.08	2.14	−	−	−
**1b**	Gly	0.47	−0.39	−0.03	2.54	20.5	−	2.21
**2b**	L-Ala	0.85	−0.18	−0.03	1.11	6.5	−	2.28
**3b**	L-Val	0.79	0.11	−	2.1	7.6	2.4	2.23
**4b**	L-Ser	0.74	0.07	−0.03	2.27	8.0	−	2.36
**5b**	D-Ser	0.74	0.08	−0.03	2.38	8.1	1.4	2.29
**6b**	L-Thr	0.82	0.07	−0.03	2.26	7.8	−	2.21
**7b**	L-Met	0.71	−	−0.01	9.85	7.5	−	2.28
**8b**	L-Tyr	0.73	0.16	−	2.27	8.5	−	2.04
**9b**	L-Lys	0.79	−0.17	−0.05	2.35	−	−	1.85
**10b**	L-S-Me-Cys	0.78	0.11	−	2.46	8.8	−	1.97
**11b**	N^ε^-Lys	0.72	−	−0.02	2.49	−	−	1.85
**12b**	β-Ala	0.41	−0.09	0.03	2.55	7.1	−	−

* Signal of mini-form is overlapped by the main form signal.

## Data Availability

Data are contained within the article or Supplementary Materials.

## Supplementary Materials

The following supporting information can be downloaded at: https://www.mdpi.com/article/10.3390/ijms24119697/s1.
