# Supplementary material for: Intramolecular Hydrogen Bonding in N6-Substituted 2-Chloroadenosines: Evidence from NMR Spectroscopy
Source: Int J Mol Sci. 2023 Jun 2;24(11):9697. doi: 10.3390/ijms24119697 (PMC10253998; doi:10.3390/ijms24119697)
Supplement: Supplementary file 1 [file ijms-24-09697-s001.zip › ijms-2427260-supplementary.pdf]

Supplementary Materials

Intramolecular Hydrogen Bonding in N⁶-Substituted 2-Chloroadenosines: Evidence from NMR Spectroscopy

Maria Ya. Berzina [1,*], Barbara Z. Eletskaya [1], Alexei L. Kayushin [1], Elena V. Dorofeeva [1], Olga I. Lutonina [1], Ilya V. Fateev [1], Olga N. Zhavoronkova [1], Arthur R. Bashorin [1], Alexandra O. Arnautova [1], Olga S. Smirnova [1], Konstantin V. Antonov [1], Alexander S. Paramonov [1], Maxim A. Dubinnyi [1,2], Roman S. Esipov [1], Anatoly I. Miroshnikov [1] and Irina D. Konstantinova [1,*]

[1] Shemyakin and Ovchinnikov Institute of Bioorganic Chemistry, Russian Academy of Sciences, Miklukho-Maklaya St. 16/10, 117997 GSP, Moscow B-437, Russian Federation; antonov.kant@yandex.ru (K.V.A.)

[2] Moscow Institute of Physics and Technology (State University), 9 Institutskiy per., Dolgoprudny, Moscow Region, 141700, Russian Federation

* M.Ya.B. berzina_maria@mail.ru; I.D.K. kid1968@yandex.ru

Content	Page number
Figure S1. The ^{1}H NMR spectrum of 9-(2',3',5'-tri-O-acetyl-β-D-ribofuranosyl)-2-chloro-6-(N$^{\alpha}$-L-valinyl)-purine, methyl ester (**3a**)	3
Figure S2. The ^{13}C NMR spectrum of 9-(2',3',5'-tri-*O*-acetyl-*β*-*D*-ribofuranosyl)-2-chloro-6-(N$^{\alpha}$-L-valinyl)-purine, methyl ester (**3a**)	4
Figure S3. The ^{15}N HSQC NMR spectrum of 9-(2',3',5'-tri-*O*-acetyl-*β*-*D*-ribofuranosyl)-2-chloro-6-(N$^{\alpha}$-L-valinyl)-purine, methyl ester (**3a**)	4
Figure S4. The ^{15}N HMBC NMR spectrum of 9-(2',3',5'-tri-*O*-acetyl-*β*-*D*-ribofuranosyl)-2-chloro-6-(N$^{\alpha}$-L-valinyl)-purine, methyl ester (**3a**)	5
Figure S5. The ^{1}H NMR spectrum of 9-*β*-*D*-ribofuranosyl-2-chloro-6-(N$^{\alpha}$-L-valinylamido)-purine (**3b**)	7
Figure S6. The ^{13}C NMR spectrum of 9-*β*-*D*-ribofuranosyl-2-chloro-6-(N$^{\alpha}$-L-valinylamido)-purine (**3b**)	7
Figure S7. The fragment of ^{13}C HSQC NMR spectrum of 9-*β*-*D*-ribofuranosyl-2-chloro-6-(N$^{\alpha}$-L-valinylamido)-purine (**3b**)	8
Figure S8. The fragment of ^{13}C HMBC NMR spectrum of 9-*β*-*D*-ribofuranosyl-2-chloro-6-(N$^{\alpha}$-L-valinylamido)-purine (**3b**)	8
Figure S9. The fragment of ^{15}N HSQC NMR spectrum of 9-*β*-*D*-ribofuranosyl-2-chloro-6-(N$^{\alpha}$-L-valinylamido)-purine (**3b**) [1]	9
Figure S10. The fragment of ^{15}N HMBC NMR spectrum of 9-*β*-*D*-ribofuranosyl-2-chloro-6-(N$^{\alpha}$-L-valinylamido)-purine (**3b**)	9
Figure S11. The ^{1}H NMR spectrum of 9-*β*-*D*-ribofuranosyl-2-chloro-6-(N$^{\alpha}$-L-valinylamido)-purine (**3b**)	11
Figure S12. The ^{13}C NMR spectrum of 9-*β*-*D*-ribofuranosyl-2-chloro-6-(N$^{\alpha}$-L-valinylamido)-purine (**3b**)	11
Figure S13. The fragment of ^{13}C HSQC NMR spectrum of 9-*β*-*D*-ribofuranosyl-2-chloro-6-(N$^{\alpha}$-L-valinylamido)-purine (**3b**)	12
Figure S14. The fragment of ^{15}N HSQC NMR spectrum of 9-*β*-*D*-ribofuranosyl-2-chloro-6-(N$^{\alpha}$-L-valinylamido)-purine (**3b**)	12
Figure S15. The fragment of ^{15}N HMBC NMR spectrum of 9-*β*-*D*-ribofuranosyl-2-chloro-6-(N$^{\alpha}$-L-valinylamido)-purine (**3b**)	13

Figure S16. The COSY NMR spectrum of 9-*β*-*D*-ribofuranosyl-2-chloro-6-(N^{α}-L-valinylamido)-purine (**3b**)	13
Figure S17. The ^{1}H NMR spectrum of 6-chloro-4-(N^{α}-L-valinyl)-pyrazolo[3,4-d]pyrimidine methyl ester (**16**)	15
Figure S18. The ^{13}C NMR spectrum of 6-chloro-4-(N^{α}-L-valinyl)-pyrazolo[3,4-d]pyrimidine methyl ester (**16**)	15
Figure S19. The fragment of ^{15}N HSQC NMR spectrum of 6-chloro-4-(N^{α}-L-valinyl)-pyrazolo[3,4-d]pyrimidine methyl ester (**16**)	16
Figure S20. The fragment of ^{15}N HMBC NMR spectrum of 6-chloro-4-(N^{α}-L-valinyl)-pyrazolo[3,4-d]pyrimidine methyl ester (**16**)	16
Figure S21. The ^{1}H NMR spectrum of 6-chloro-1-*β*-*D*-ribofuranosyl-4-(N^{α}-L-valinyl)-pyrazolo[3,4-d]pyrimidine methyl ester (**13**)	18
Figure S22. The ^{13}C NMR spectrum of 6-chloro-1-*β*-*D*-ribofuranosyl-4-(N^{α}-L-valinyl)-pyrazolo[3,4-d]pyrimidine methyl ester (**13**)	18
Figure S23. The fragment of ^{15}N HSQC NMR spectrum of 6-chloro-1-*β*-*D*-ribofuranosyl-4-(N^{α}-L-valinyl)-pyrazolo[3,4-d]pyrimidine methyl ester (**13**)	19
Figure S24. The fragment of ^{15}N HMBC NMR spectrum of 6-chloro-1-*β*-*D*-ribofuranosyl-4-(N^{α}-L-valinyl)-pyrazolo[3,4-d]pyrimidine methyl ester (**13**)	19
Figure S25. The ^{1}H NMR spectrum of 9-(2',3',5'-tri-O-acetyl-*β*-*D*-ribofuranosyl)-2-chloro-6-*tert*-butylamino-purine (**18**)	21
Figure S26. The ^{13}C NMR spectrum of 9-(2',3',5'-tri-*O*-acetyl-*β*-*D*-ribofuranosyl)-2-chloro-6-*tert*-butylamino-purine (**18**)	21
Figure S27. The fragment of ^{13}C HSQC NMR spectrum of 9-(2',3',5'-tri-*O*-acetyl-*β*-*D*-ribofuranosyl)-2-chloro-6-*tert*-butylamino-purine (**18**)	22
Figure S28. The fragment of ^{15}N HSQC NMR spectrum of 9-(2',3',5'-tri-*O*-acetyl-*β*-*D*-ribofuranosyl)-2-chloro-6-*tert*-butylamino-purine (**18**)	22
Figure S29. The fragment of ^{15}N HMBC NMR spectrum of 9-(2',3',5'-tri-*O*-acetyl-*β*-*D*-ribofuranosyl)-2-chloro-6-*tert*-butylamino-purine (**18**)	23
Figure S30. The ^{1}H NMR spectrum of 9-*β*-*D*-ribofuranosyl-2-chloro-6-*tert*-butylamino-purine (**14**)	24
Figure S31. The ^{13}C NMR spectrum of 9-*β*-*D*-ribofuranosyl-2-chloro-6-*tert*-butylamino-purine (**14**)	24
Figure S32. The fragment of ^{15}N HSQC NMR spectrum of 9-*β*-*D*-ribofuranosyl-2-chloro-6-*tert*-butylamino-purine (**14**)	25
Figure S33. The fragment of ^{15}N HMBC NMR spectrum of 9-*β*-*D*-ribofuranosyl-2-chloro-6-*tert*-butylamino-purine (**14**)	25
Figure S34. Full assignment of signals of the main and mini-form of compound **3b**	26
References	26

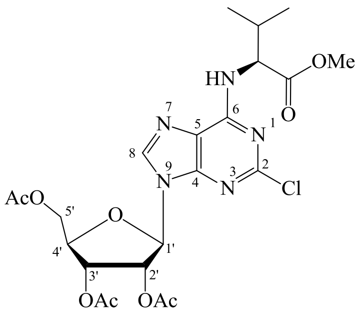

3a

Data from reference [1]
9-(2',3',5'-Tri-*O*-acetyl-*β*-*D*-ribofuranosyl)-2-chloro-6-(N^{α}-L-valinyl)-purine, methyl ester (**3a**)

^{1}H NMR (700 MHz, DMSO-d_6, *J*, Hz, 30 °C): δ 8.63 and 8.50 (2 d, *J* = 6.6 Hz and *J* = 6.6 Hz, 0.63 and 0.28 H, NH), 8.47 and 8.40 (2 s, 0.66 and 0.34 H, H8), 6.18 and 6.16 (d, *J* = 5.2 Hz, and m, 1 H, $H_{1'}$), 5.90 (t, *J* = 5.4 Hz, 1H, $H_{2'}$), 5.59 (m, 1 H, $H_{3'}$), 5.50 and 4.46 (2 m, 0.32 and 0.65 H, $^{\alpha}$CH-Val), 4.39 (m, 2 H, $H_{5'a}$ and $H_{4'}$), 4.27 (m, 1 H, $H_{5'b}$), 3.65 and 3.64 (2 sign, 3H, OCH_3), 2.29 (m, 1 H, $^{\beta}$CH-Val), 2.12 (s, 3 H, CH_3CO-3'), 2.05 (s, 3 H, CH_3CO-2'), 2.02 (s, 3 H, CH_3CO-5'), 1.01 and 0.96 ppm (2 d, *J* = 6.8 Hz and *J* = 6.7 Hz, 6 H, 2 CH_3-Val).
^{13}C NMR (176 MHz, DMSO-d_6, 30 °C): δ 171.75 (C̲O-OCH_3), 169.90 (O-$CO^{5'}$), 169.30 (O-$CO^{3'}$), 169.16 (O-$CO^{2'}$), 154.81 (C6), 152.84 (C2), 149.68 (C4), 140.46 (C8), 118.47 (C5), 85.36 ($C_{1'}$), 79.51 ($C_{4'}$), 72.03 ($C_{2'}$), 69.85 ($C_{3'}$), 62.60 ($C_{5'}$), 60.53 miniform and 59.31 ($^{\alpha}$CH-Val), 51.60 (CO-OC̲H_3), 29.26 ($^{\beta}$CH-Val), 20.35 (C̲H_3-$CO^{5'}$), 20.25 (C̲H_3-$CO^{3'}$), 20.09 (C̲H_3-$CO^{2'}$), 19.11 (CH_3-Val), 18.93 ppm (CH_3-Val).

^{15}N NMR (71 MHz, DMSO-d_6, 30 °C): δ 242.7 (N7), 167.1 (N9), 99.3 miniform and 95.2 ppm (NH).

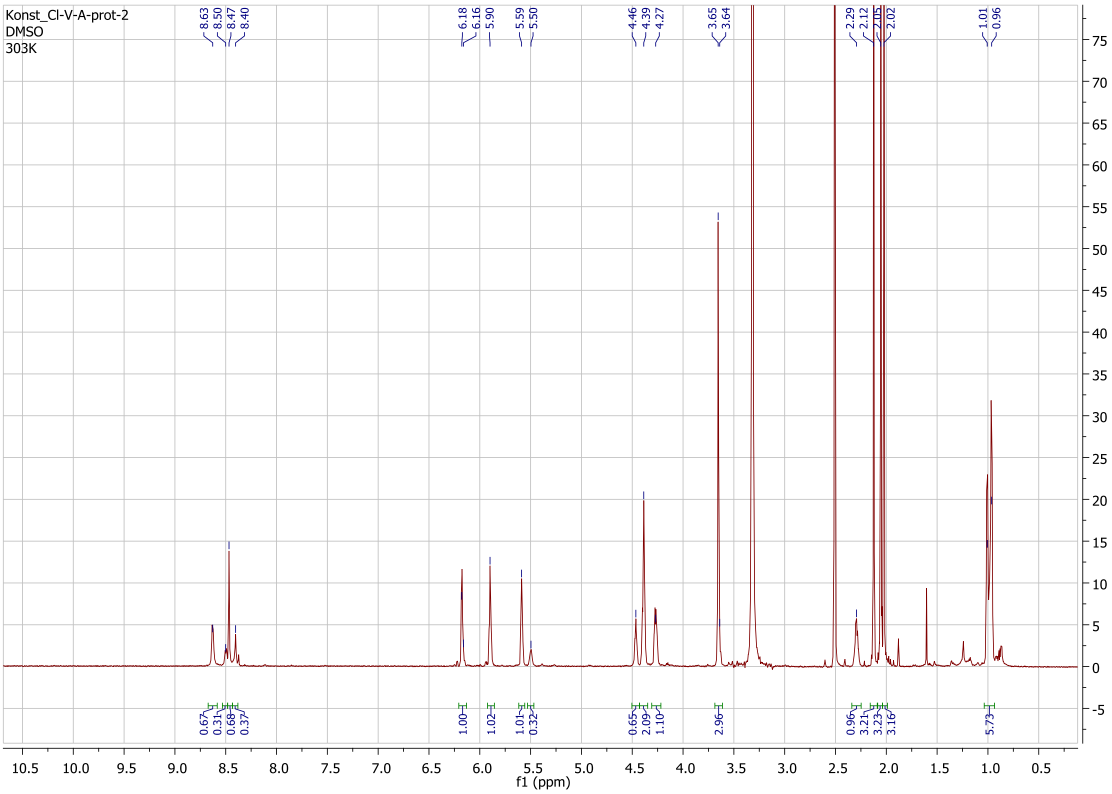

Figure S1. The ^{1}H NMR spectrum of 9-(2',3',5'-tri-O-acetyl-*β*-*D*-ribofuranosyl)-2-chloro-6-(N^{α}-L-valinyl)-purine, methyl ester (**3a**) [1]

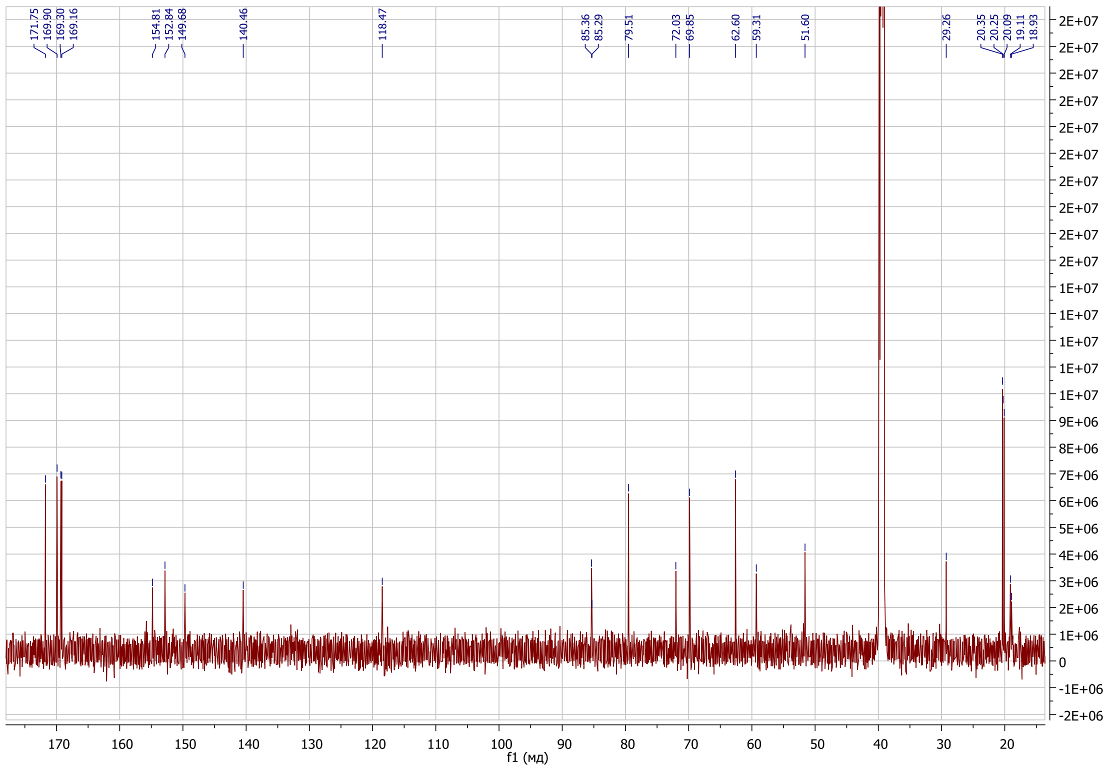

Figure S2. The ^{13}C NMR spectrum of 9-(2',3',5'-tri-*O*-acetyl-*β*-*D*-ribofuranosyl)-2-chloro-6-(N$^{\alpha}$-L-valinyl)-purine, methyl ester (**3a**) [1]

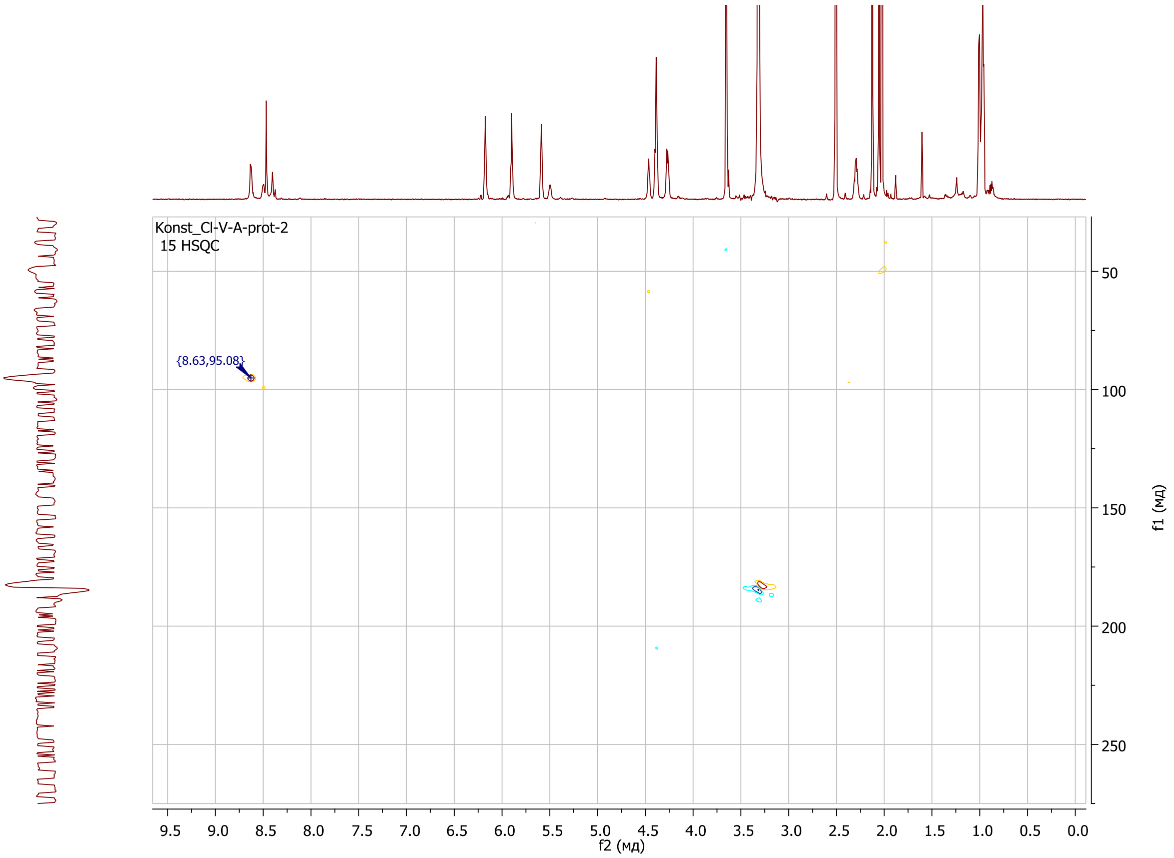

Figure S3. The ^{15}N HSQC NMR spectrum of 9-(2',3',5'-tri-*O*-acetyl-*β*-*D*-ribofuranosyl)-2-chloro-6-(N$^{\alpha}$-L-valinyl)-purine, methyl ester (**3a**) [1]

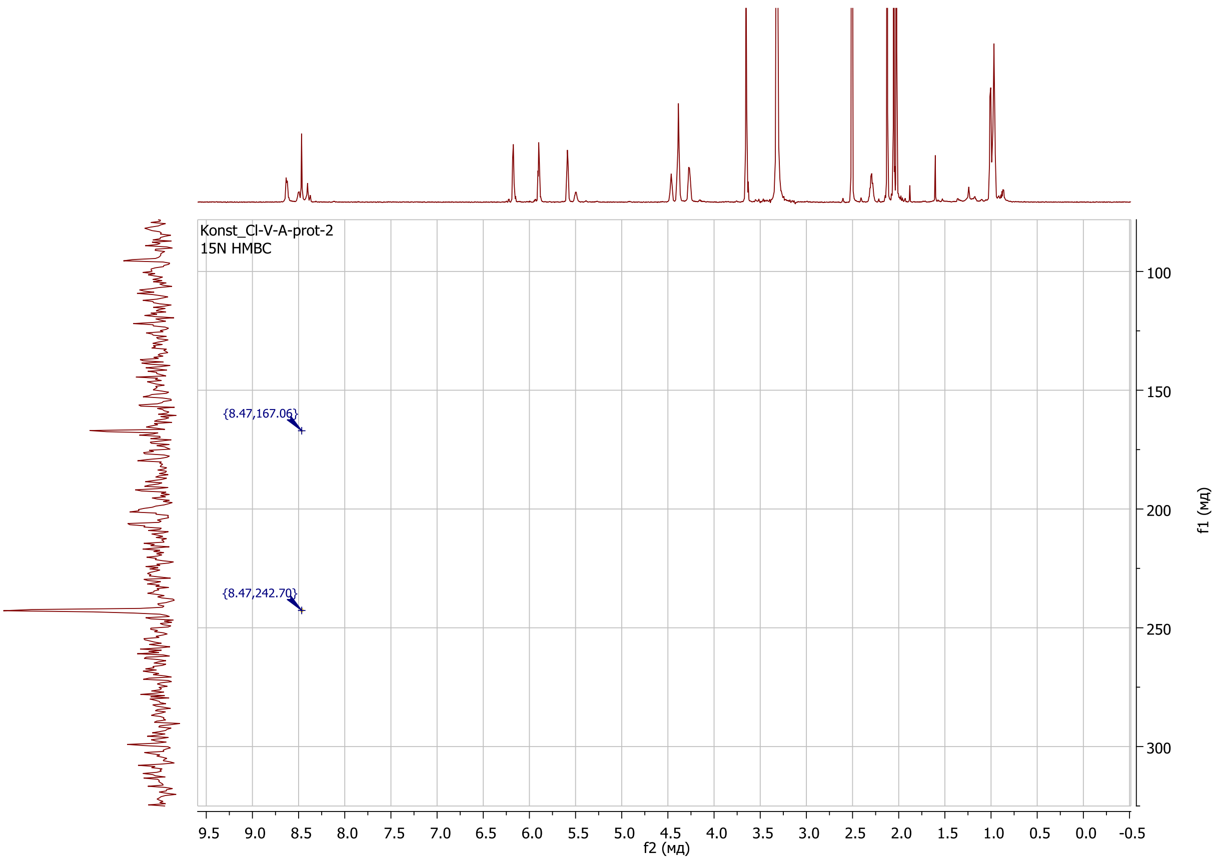

Figure S4. The ^{15}N HMBC NMR spectrum of 9-(2',3',5'-tri-*O*-acetyl-*β*-*D*-ribofuranosyl)-2-chloro-6-(N$^{\alpha}$-L-valinyl)-purine, methyl ester (**3a**) [1]

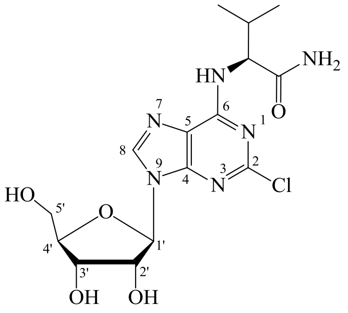<br><br>3b	Data from reference [1]<br>9-*β*-*D*-ribofuranosyl-2-chloro-6-(N$^{\alpha}$-L-valinylamido)-purine (**3b**).<br><br>^{1}H NMR (700 MHz, DMSO-d_6, *J*, Hz, 30 °C): δ 8.45 (s, 1 H, H 8), 7.85 and 7.52 (m and d, *J* = 8.4 Hz, 0.19 and 0.81 H, NH), 7.55, 7.38, 7.20, and 7.14 (4 s, 0.60, 0.17, 0.71, and 0.22 H, NH_2), 5.85 (d, *J* = 5.8 Hz, 1 H, $H_{1'}$), 5.48 (d, *J* = 6.2 Hz, 1 H, $OH_{2'}$), 5.33 and 4.53 (2 m, 0.19 and 0.70 H, $^{\alpha}$CH-Val), 5.19 (d, *J* = 5.0 Hz, 1 H, $OH_{3'}$), 5.03 (m, 1 H, $OH_{5'}$), 4.53 (m, 1 H, $H_{2'}$ and 0.70 H, $^{\alpha}$CH-Val), 4.14 (m, 1 H, $H_{3'}$), 3.95 (m, 1 H, $H_{4'}$), 3.67 (m, 1 H, $H_{5'a}$), 3.56 (m, 1 H, $H_{5'b}$), 2.19 (m, 1 H, $^{\beta}$CH-Val), 0.96 (d, *J* = 6.7 Hz, 3H, CH_3-Val), 0.93 ppm (d, *J* = 6.6 Hz, 3 H, CH_3-Val).<br>^{13}C NMR (176 MHz, DMSO-d_6, 30 °C) main form (81%): δ 172.34 ($CONH_2$), 154.72 (C6), 152.81 (C2), 149.66 (C4), 140.29 (C8), 118.51 (C5), 87.42 ($C_{1'}$), 85.63 ($C_{4'}$), 73.57 ($C_{2'}$), 70.24 ($C_{3'}$), 61.22 ($C_{5'}$), 58.88 ($^{\alpha}$CH-Val), 30.52 ($^{\beta}$CH-Val), 19.13 (CH_3-Val), 18.28 and 17.45 ppm (CH_3-Val).<br>^{13}C NMR (176 MHz, DMSO-d_6, 30 °C) miniform (19%): δ 172.49 ($CONH_2$), 155.47 (C6) 152.63 (C2), 151.89 (C4), 139.61 (C8), 117.32 (C5), 87.20 ($C_{1'}$), 85.63 ($C_{4'}$), 73.57 ($C_{2'}$), 70.24 ($C_{3'}$), 61.22 ($C_{5'}$), 61.04 ($^{\alpha}$CH-Val), 30.52 ($^{\beta}$CH-Val), 19.13 (CH_3-Val), 18.28 and 17.45 ppm (CH_3-Val).<br>^{15}N NMR (71 MHz, DMSO-d_6, 30 °C): δ 240.1 (N7), 227.1 (N1), 171.4 (N9), 106.4 (NH_2), 101.1 miniform and 93.1 ppm (NH).

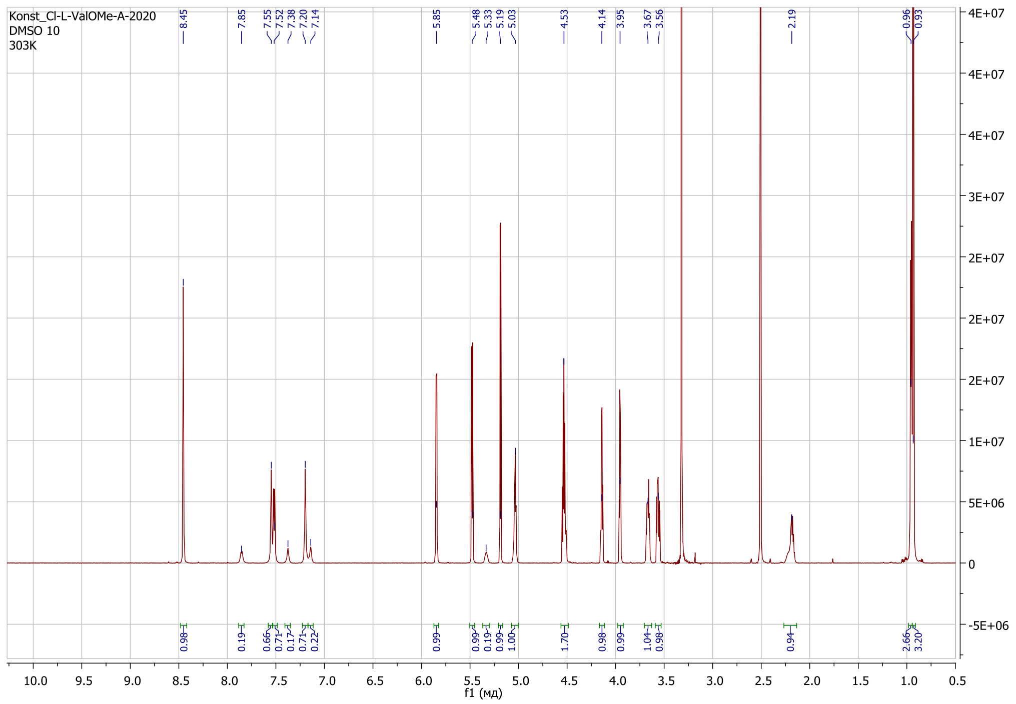

Figure S5. The ^{1}H NMR spectrum of 9-*β*-*D*-ribofuranosyl-2-chloro-6-(N$^{\alpha}$-L-valinylamido)-purine (**3b**) [1]

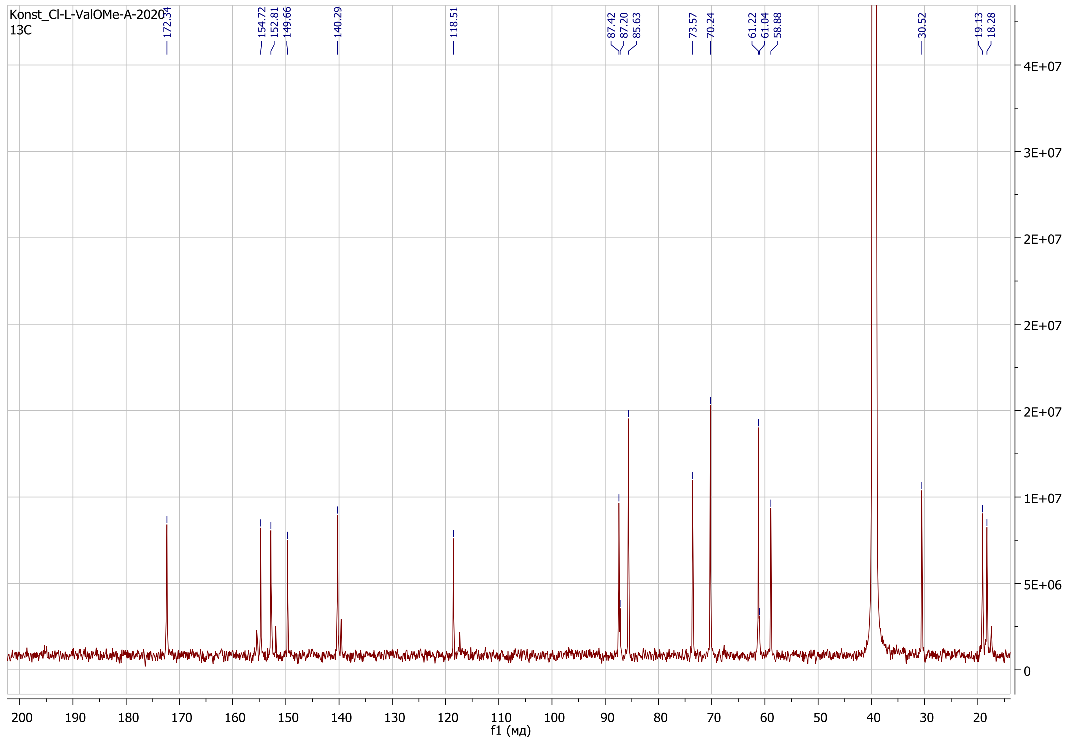

Figure S6. The ^{13}C NMR spectrum of 9-*β*-*D*-ribofuranosyl-2-chloro-6-(N$^{\alpha}$-L-valinylamido)-purine (**3b**) [1]

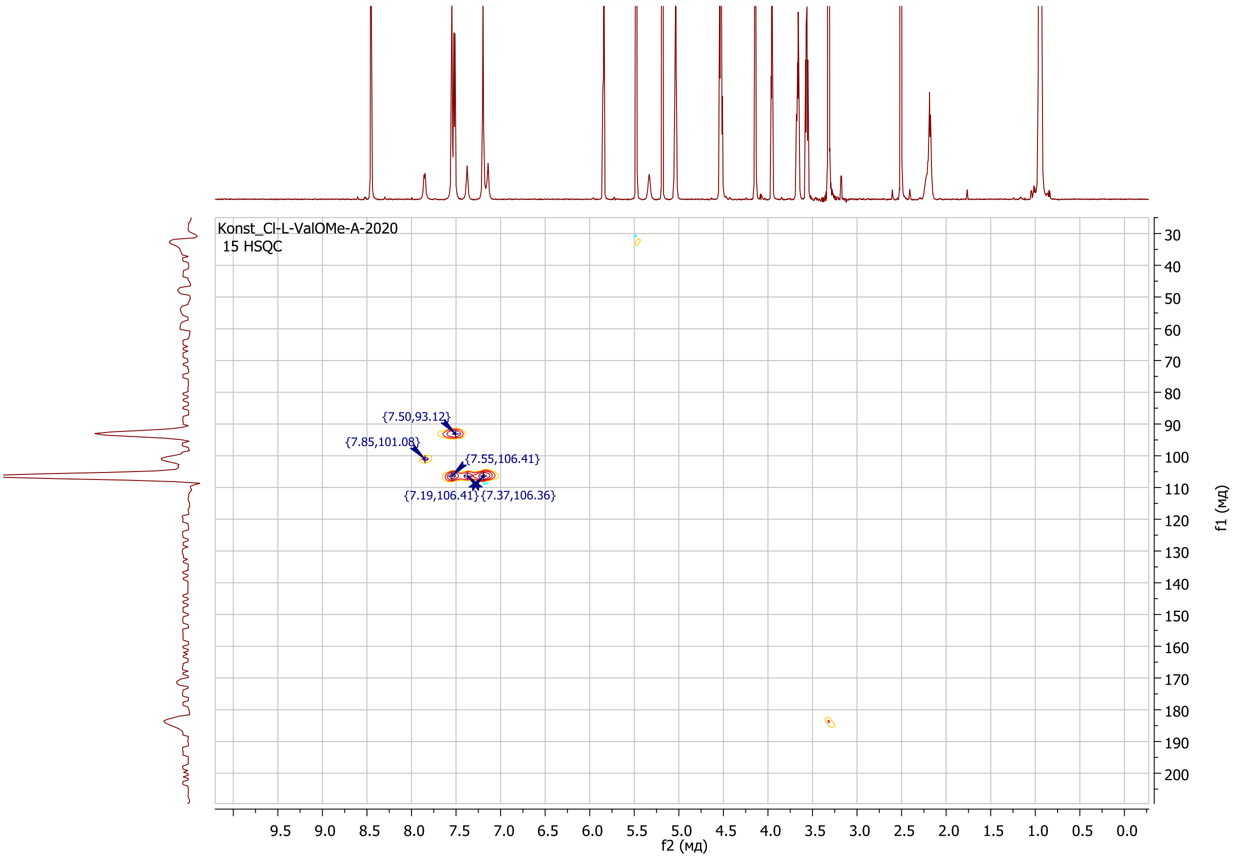

Figure S7. The fragment of ^{13}C HSQC NMR spectrum of 9-*β*-*D*-ribofuranosyl-2-chloro-6-(N$^{\alpha}$-L-valinylamido)-purine (**3b**) [1]

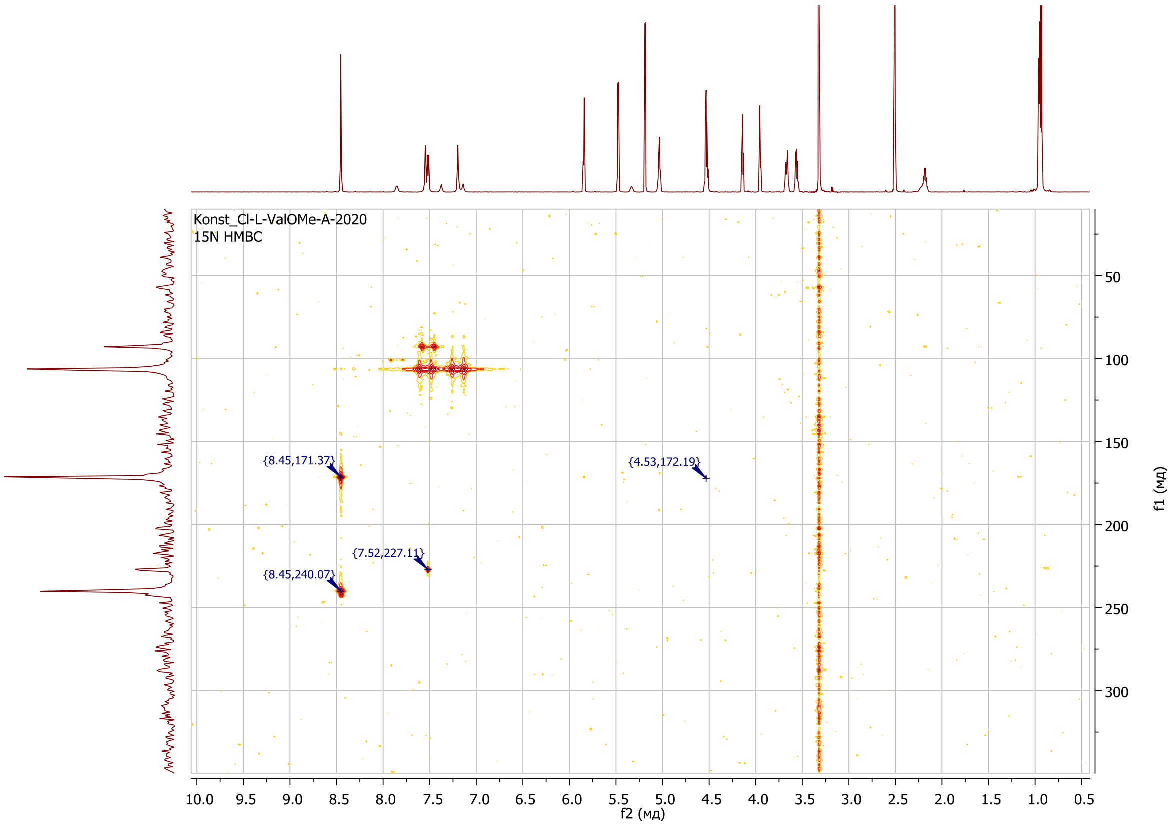

Figure S8. The fragment of ^{13}C HMBC NMR spectrum of 9-*β*-*D*-ribofuranosyl-2-chloro-6-(N$^{\alpha}$-L-valinylamido)-purine (**3b**) [1]

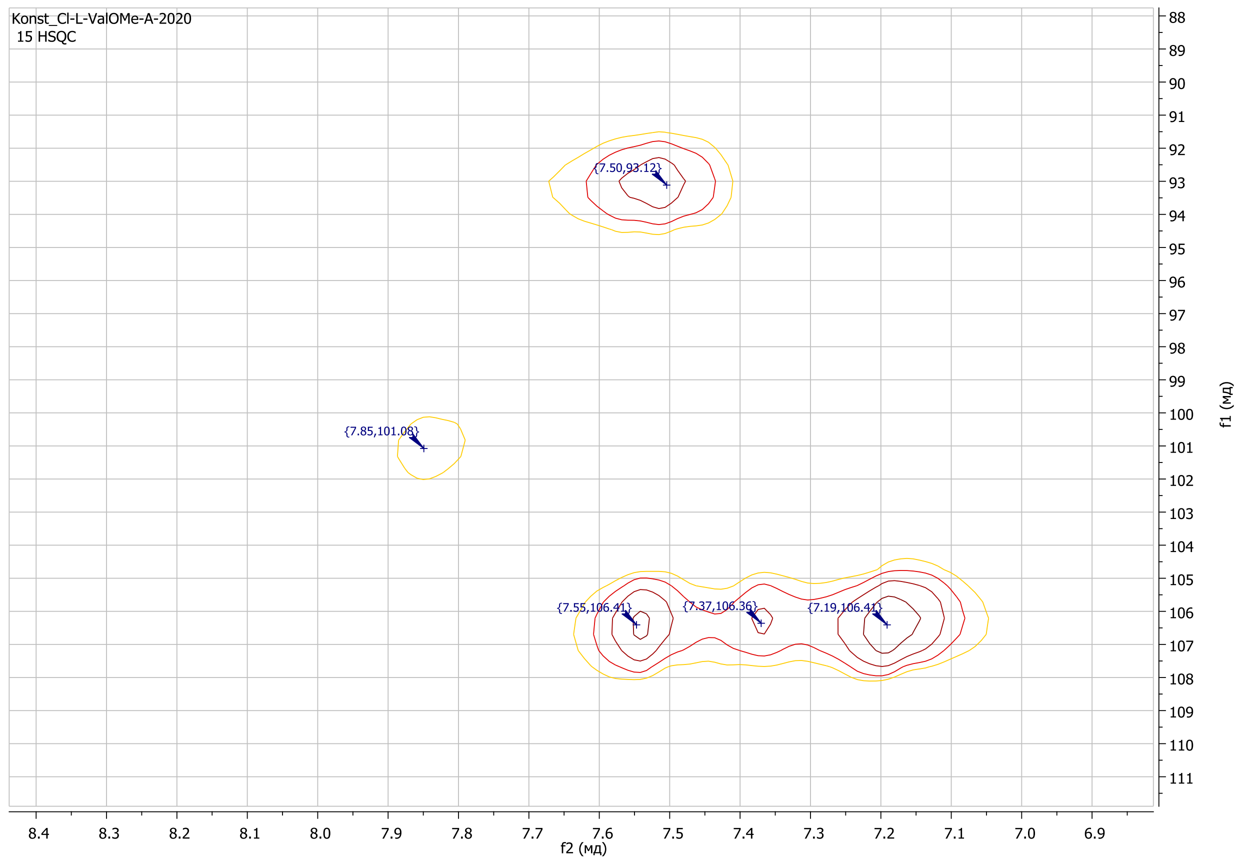

Figure S9. The fragment of ^{15}N HSQC NMR spectrum of 9-*β*-*D*-ribofuranosyl-2-chloro-6-(N^{α}-L-valinylamido)-purine (**3b**) [1]

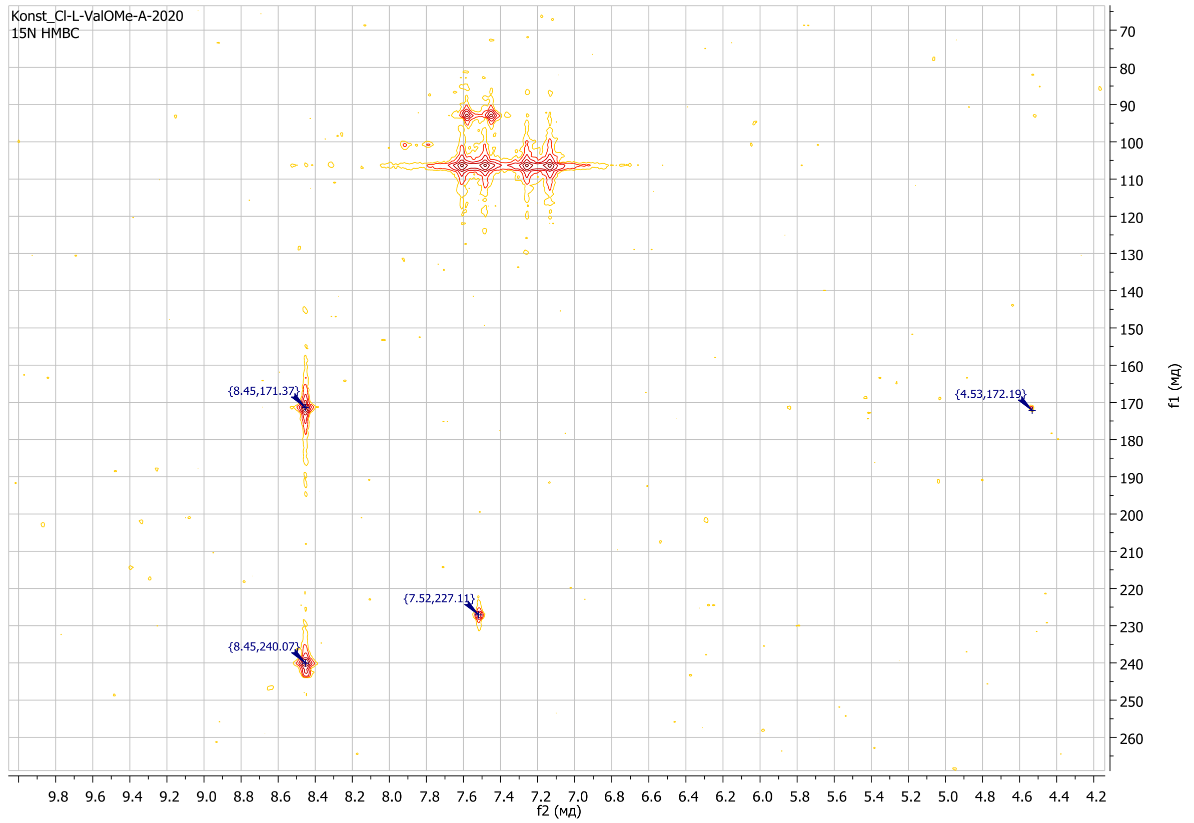

Figure S10. The fragment of ^{15}N HMBC NMR spectrum of 9-*β*-*D*-ribofuranosyl-2-chloro-6-(N^{α}-L-valinylamido)-purine (**3b**) [1]

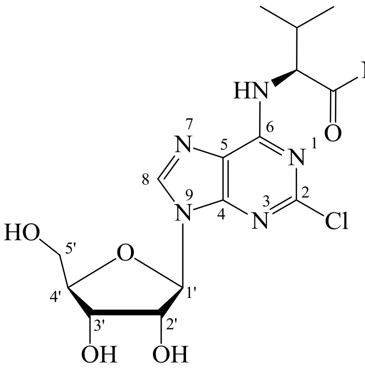<br>**3b**	9-*β*-*D*-ribofuranosyl-2-chloro-6-(N^{α}-L-valinylamido)-purine (**3b**)<br><br>^{1}H NMR (800 MHz, DMSO-d_6, *J*, Hz, 30 °C): δ 8.42 (br.s. and s, 1 H, H 8), 7.78 and 7.67 (2 m, 0.19 and 0.81 H, NH), 7.67, 7.53 and 7.19 (3 s, 0.77, 0.17, and 0.91 H, NH_2), 5.90 (d, *J* = 5.8 Hz, 1 H, $H_{1'}$), 5.67 (d, *J* = 6.2 Hz, 1 H, $OH_{2'}$), 5.38 (s, 1 H, $OH_{3'}$), 5.33 and 4.54 (2 m, 0.20 and 0.77 H, $^{\alpha}$CH-Val), 5.25 (m, 1 H, $OH_{5'}$), 4.59 (m, 1 H, $H_{2'}$), 4.23 (m, 1 H, $H_{3'}$), 4.04 (m, 1 H, $H_{4'}$), 3.72 (m, 1 H, $H_{5'a}$), 3.63 (m, 1 H, $H_{5'b}$), 2.16 (m, 1 H, $^{\beta}$CH-Val), 0.90 (m, 6 H, CH_3-Val). ^{13}C NMR (176 MHz, DMSO-d_6, 30 °C) main form (77%): δ 172.75 ($CONH_2$), 155.26 (C6), 153.56 (C2), 150.06 (C4), 140.85 (C8), 118.95 (C5), 88.36 ($C_{1'}$), 86.25 ($C_{4'}$), 74.38 ($C_{2'}$), 70.92 ($C_{3'}$), 61.85 ($C_{5'}$), 59.57 ($^{\alpha}$CH-Val), 30.92 ($^{\beta}$CH-Val), 19.54 (CH_3-Val), 18.46 ppm (CH_3-Val). ^{13}C NMR (176 MHz, DMSO-d_6, 30 °C) miniform (20%): δ 173.95 ($CONH_2$), 155.92 (C6) 153.25 (C2), 152.31 (C4), 140.31 (C8), 117.97 (C5), 88.14 ($C_{1'}$), 74.25 ($C_{2'}$), 61.67 ($^{\alpha}$CH-Val), 31.04 ($^{\beta}$CH-Val), 19.39 (CH_3-Val), 17.94 ppm (CH_3-Val). ^{15}N NMR (71 MHz, DMSO-d_6, 30 °C): δ 242.57 miniform and 237.28 (N7), 227.62 (N1), 221.20 (N3) 171.28 (N9), 107.92 miniform and 107.95 (NH_2), 101.12 miniform and 93.53 ppm (NH).

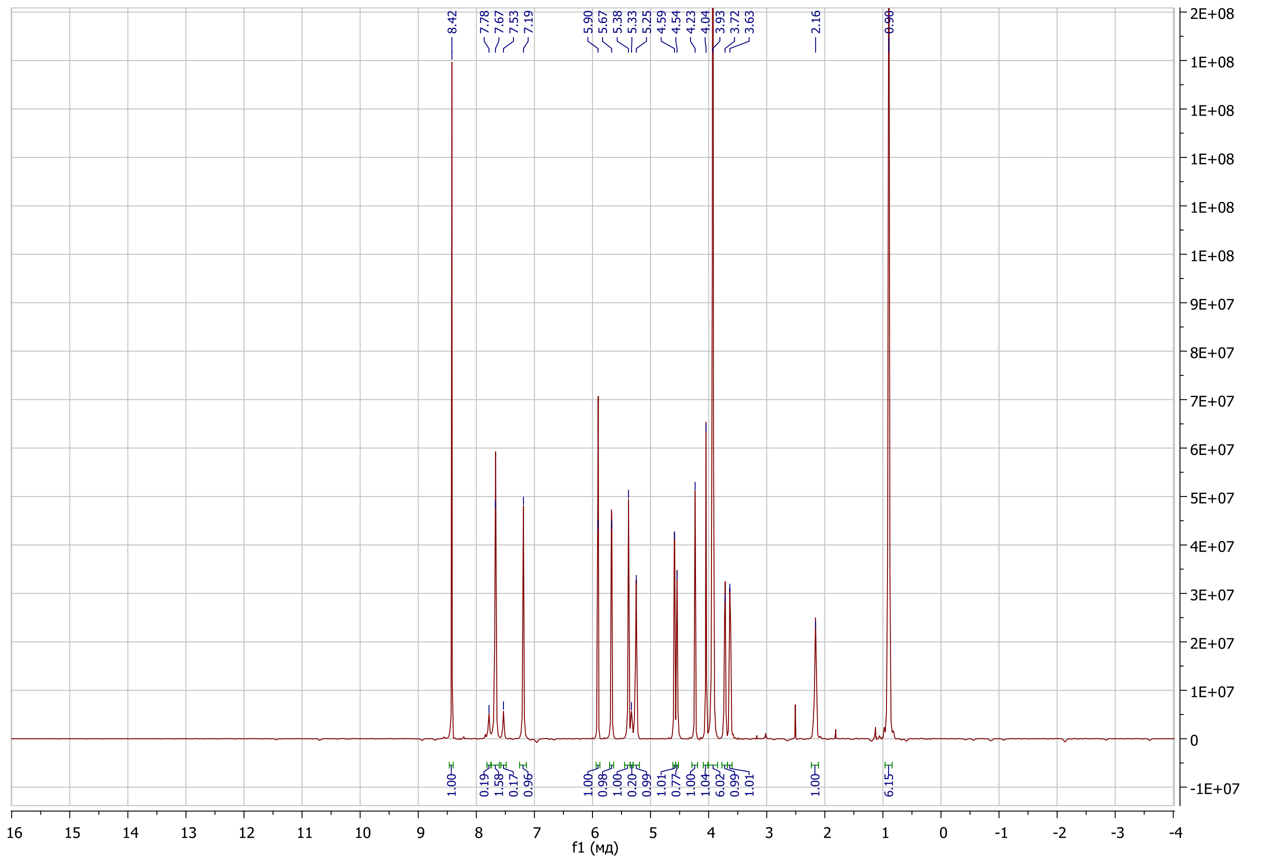

Figure S11. The ^{1}H NMR spectrum of 9-*β*-*D*-ribofuranosyl-2-chloro-6-(N$^{\alpha}$-L-valinylamido)-purine (**3b**)

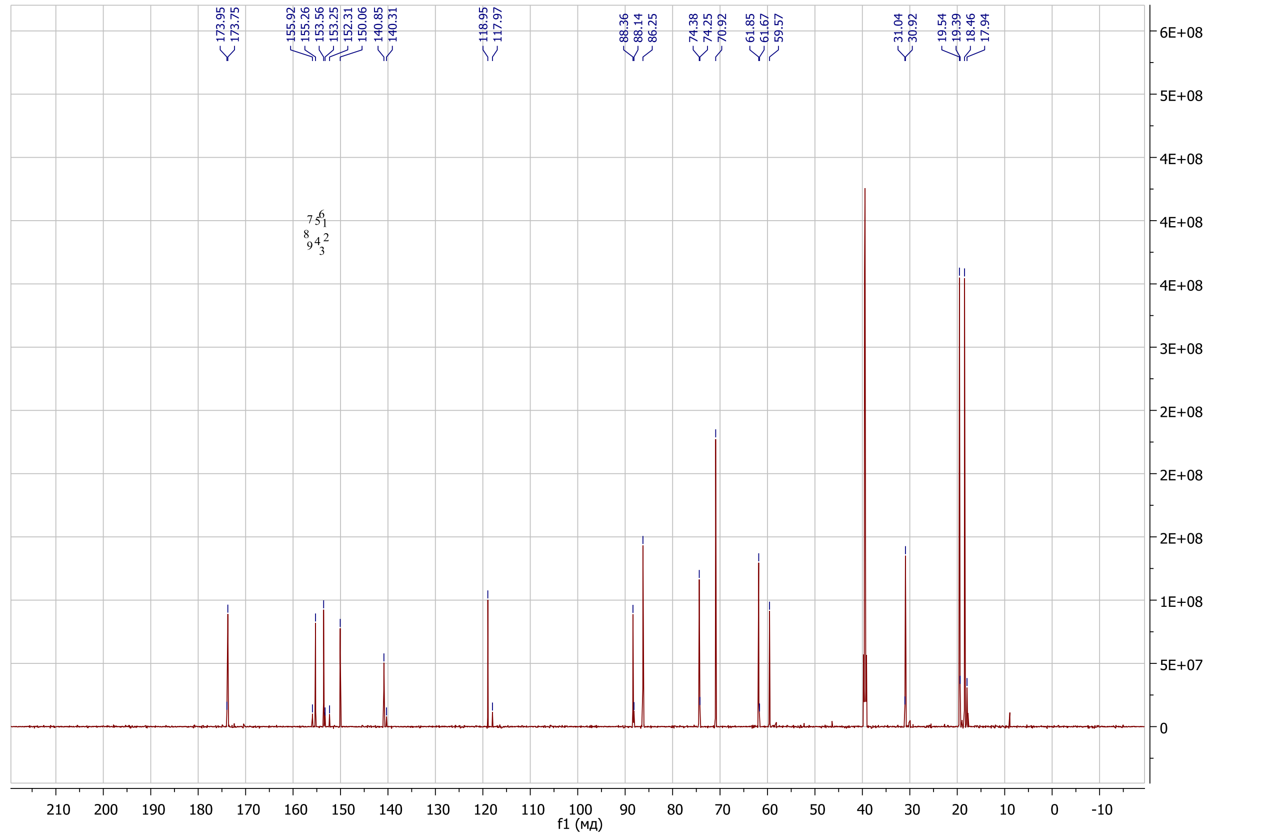

Figure S12. The ^{13}C NMR spectrum of 9-*β*-*D*-ribofuranosyl-2-chloro-6-(N$^{\alpha}$-L-valinylamido)-purine (**3b**)

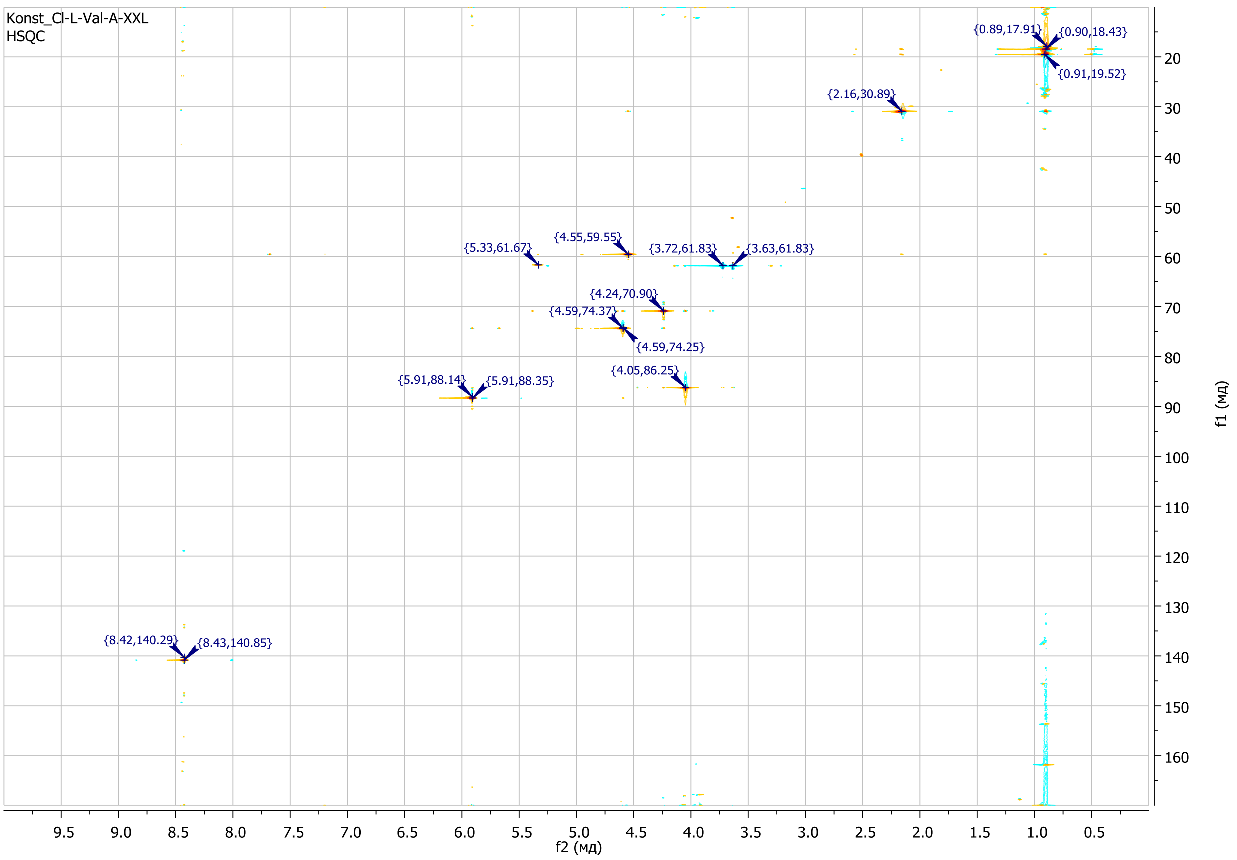

Figure S13. The fragment of ^{13}C HSQC NMR spectrum of 9-*β*-*D*-ribofuranosyl-2-chloro-6-(N$^{\alpha}$-L-valinylamido)-purine (**3b**)

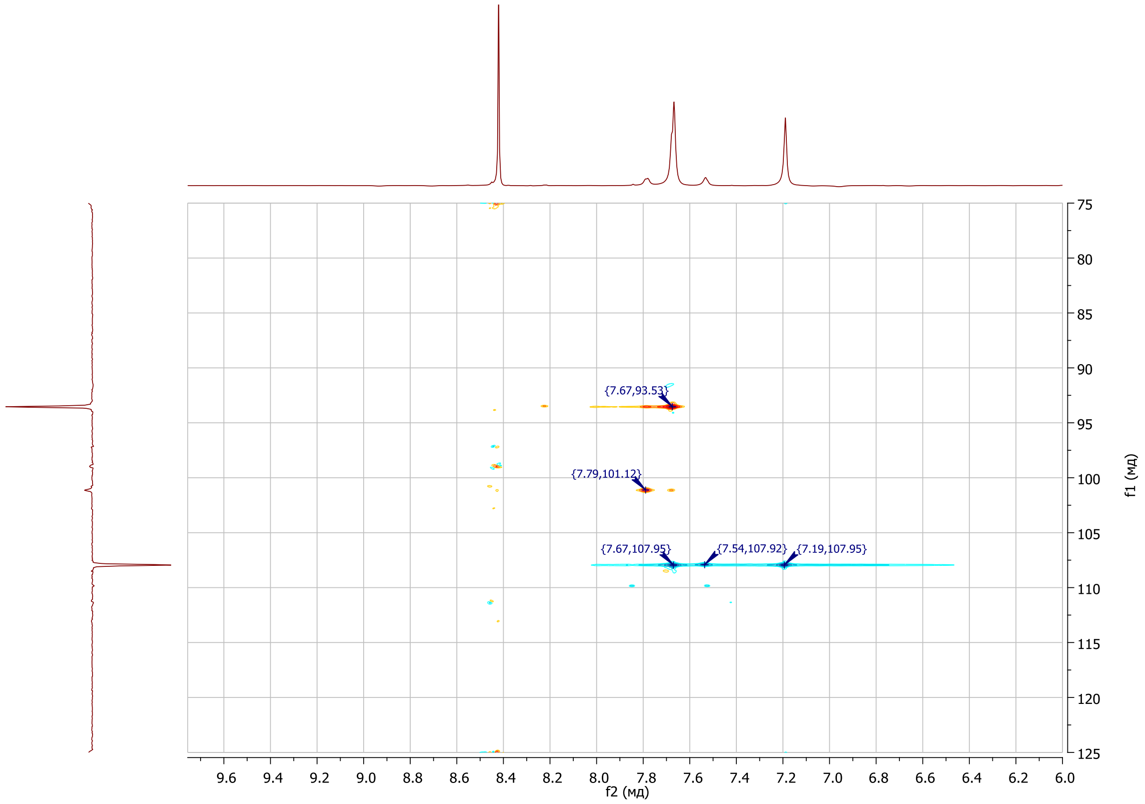

Figure S14. The fragment of ^{15}N HSQC NMR spectrum of 9-*β*-*D*-ribofuranosyl-2-chloro-6-(N$^{\alpha}$-L-valinylamido)-purine (**3b**)

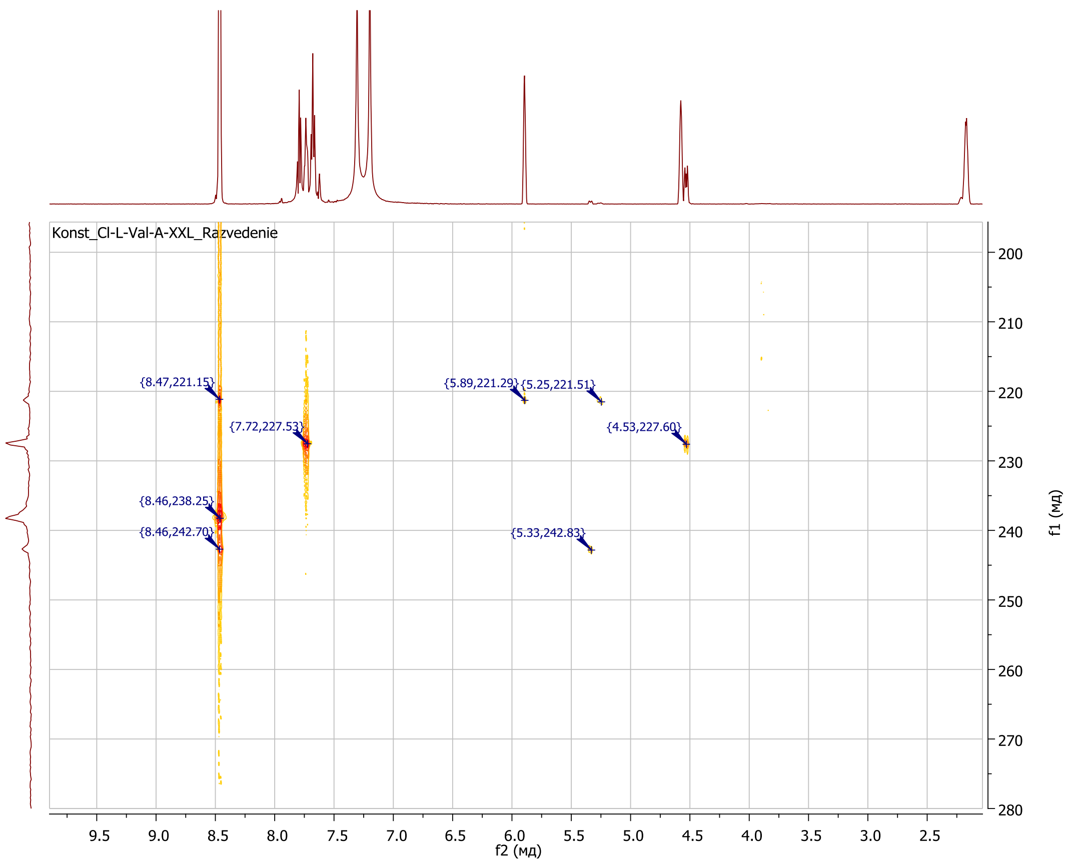

Figure S15. The fragment of ^{15}N HMBC NMR spectrum of 9-*β*-*D*-ribofuranosyl-2-chloro-6-(N$^{\alpha}$-L-valinylamido)-purine (**3b**)

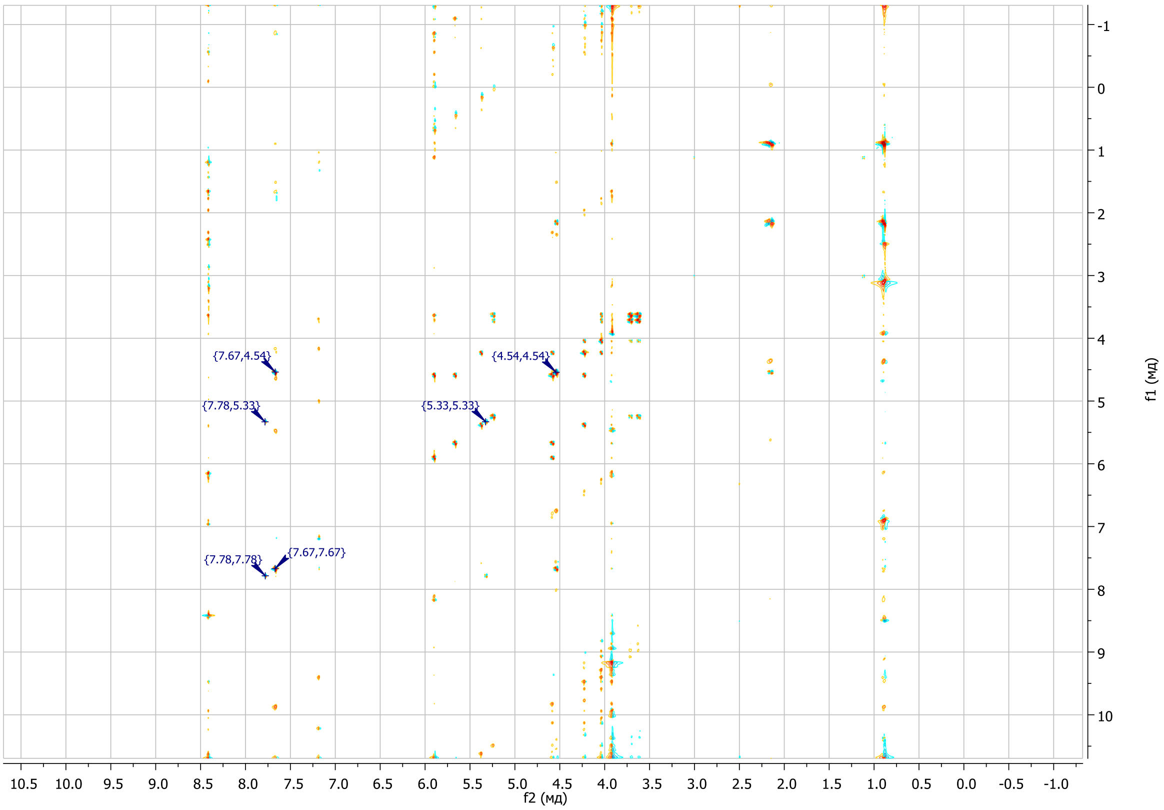

Figure S16. The COSY NMR spectrum of 9-*β*-*D*-ribofuranosyl-2-chloro-6-(N$^{\alpha}$-L-valinylamido)-purine (**3b**)

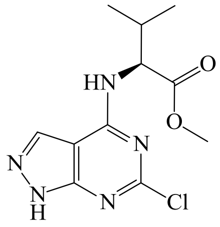 **16**	6-chloro-4-(N^{α}-L-valinyl)-pyrazolo[3,4-d]pyrimidine methyl ester (**16**)<br><br>^{1}H NMR (700 MHz, DMSO-d_6, J, Hz, 30 °C): δ 13.60 (s, 0.89 H, N1-H), 8.81 (d, J = 7.4 H, 1 H, NH-Val), 8.35 (s, 1 H, H3), 4.62 (m, 1 H, 1 H, $^{\alpha}$CH-Val), 3.69 (s, 3 H, -OCH_3), 2.21 (m, 1 H, $^{\beta}$CH-Val), 1.02 (d, J = 6.2 Hz, 3 H, CH_3), 0.98 ppm (d, J = 6.3 Hz, 3 H, CH_3). ^{13}C NMR (176 MHz, DMSO-d_6, 30 °C): δ 171.73 (<u>C</u>$OOCH_3$), 156.58 (C4), 155.35 (C6 or C7a), 133.14 (C3), 98.65 (C4a), 58.87 ($^{\alpha}$CH-Val), 51.70 (-OCH_3), 29.99 ($^{\beta}$CH-Val), 18.81 (CH_3), 18.74 ppm (CH_3). ^{15}N NMR (71 MHz, DMSO-d_6, 30 °C): δ 221.5 (N5), 190.8 (N1), 101.9 ppm (NH-Val).

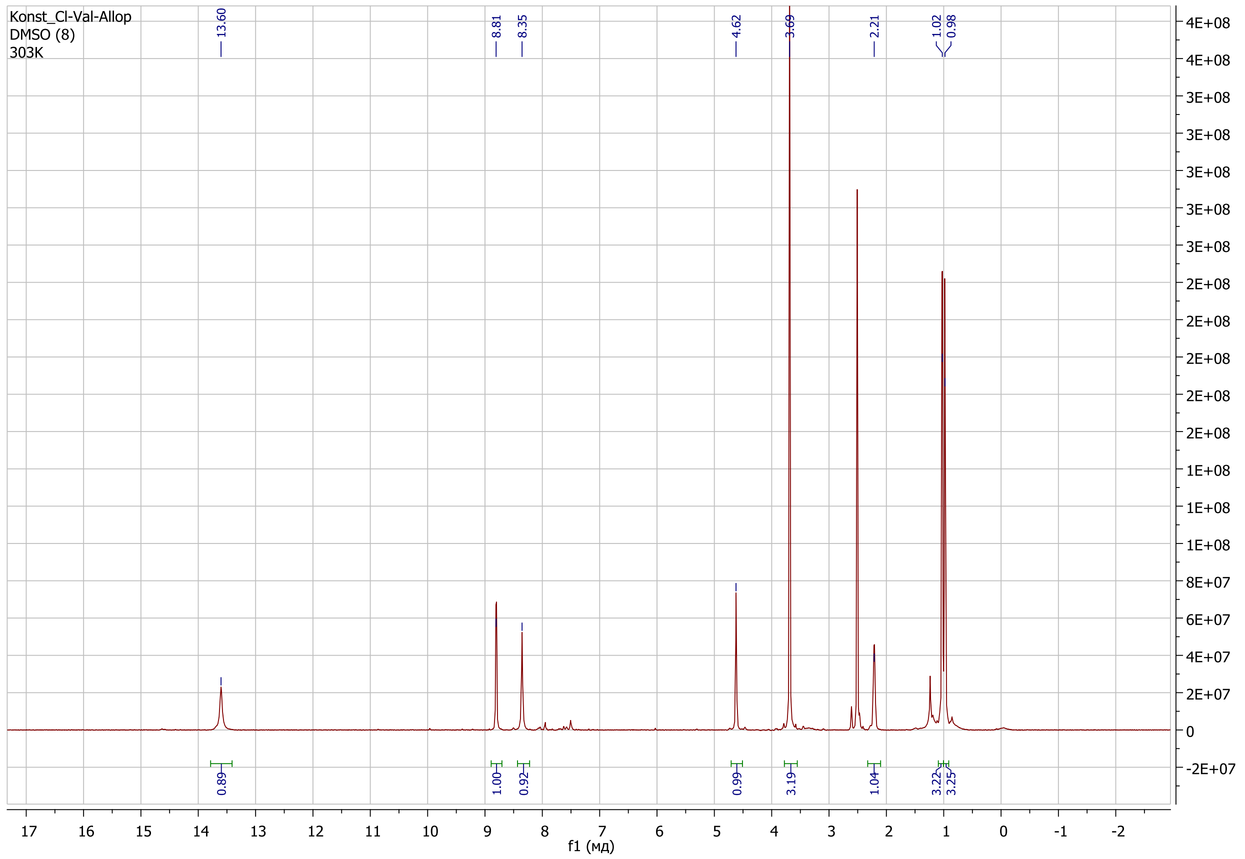

Figure S17. The ^{1}H NMR spectrum of 6-chloro-4-(N$^{\alpha}$-L-valinyl)-pyrazolo[3,4-d]pyrimidine methyl ester (**16**)

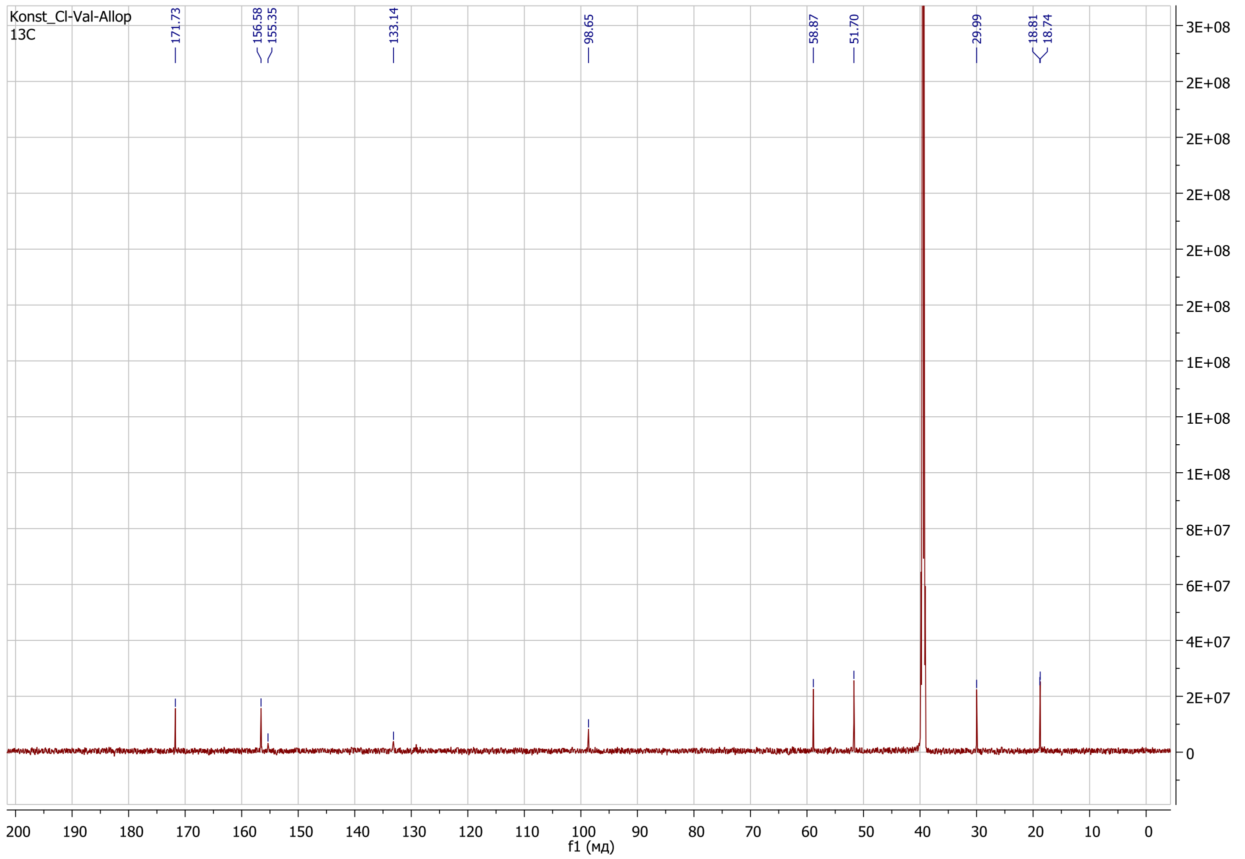

Figure S18. The ^{13}C NMR spectrum of 6-chloro-4-(N$^{\alpha}$-L-valinyl)-pyrazolo[3,4-d]pyrimidine methyl ester (**16**)

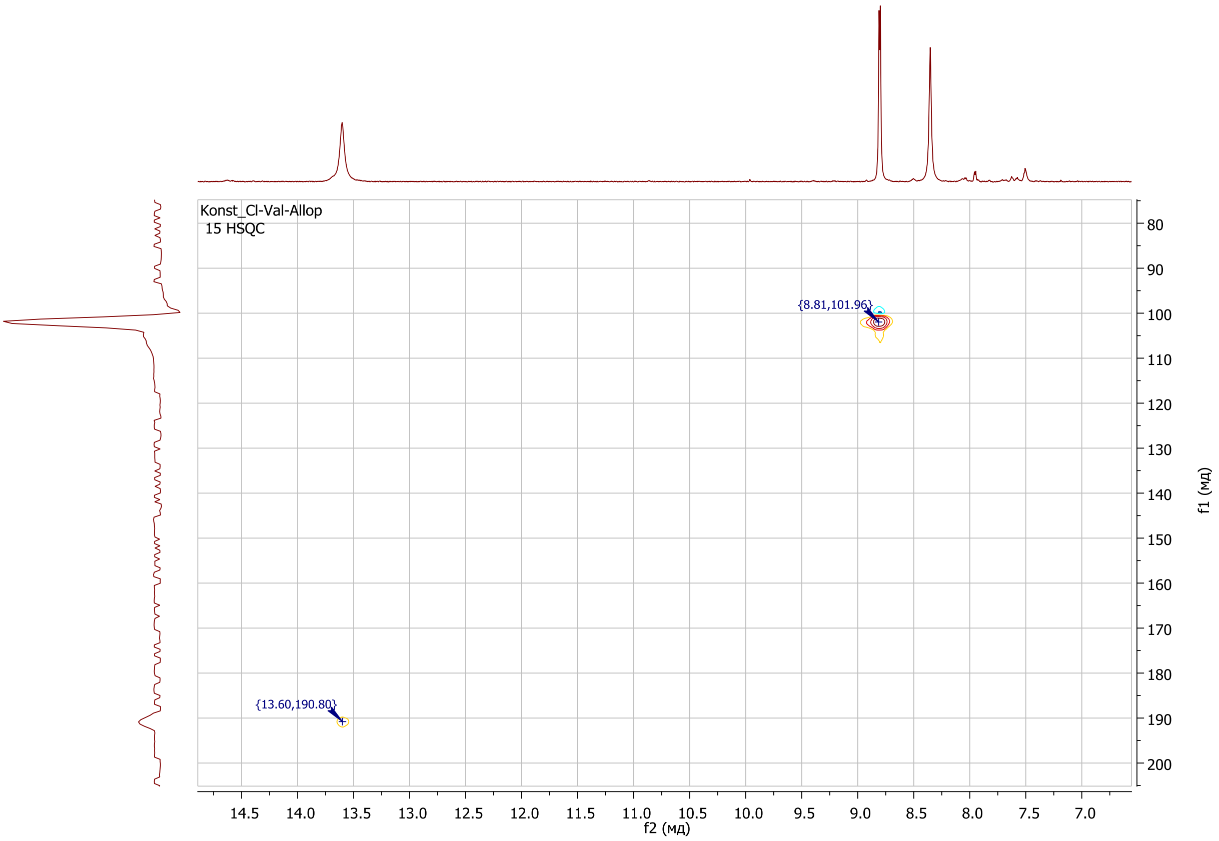

Figure S19. The fragment of ^{15}N HSQC NMR spectrum of 6-chloro-4-(N$^{\alpha}$-L-valinyl)-pyrazolo[3,4-d]pyrimidine methyl ester (**16**)

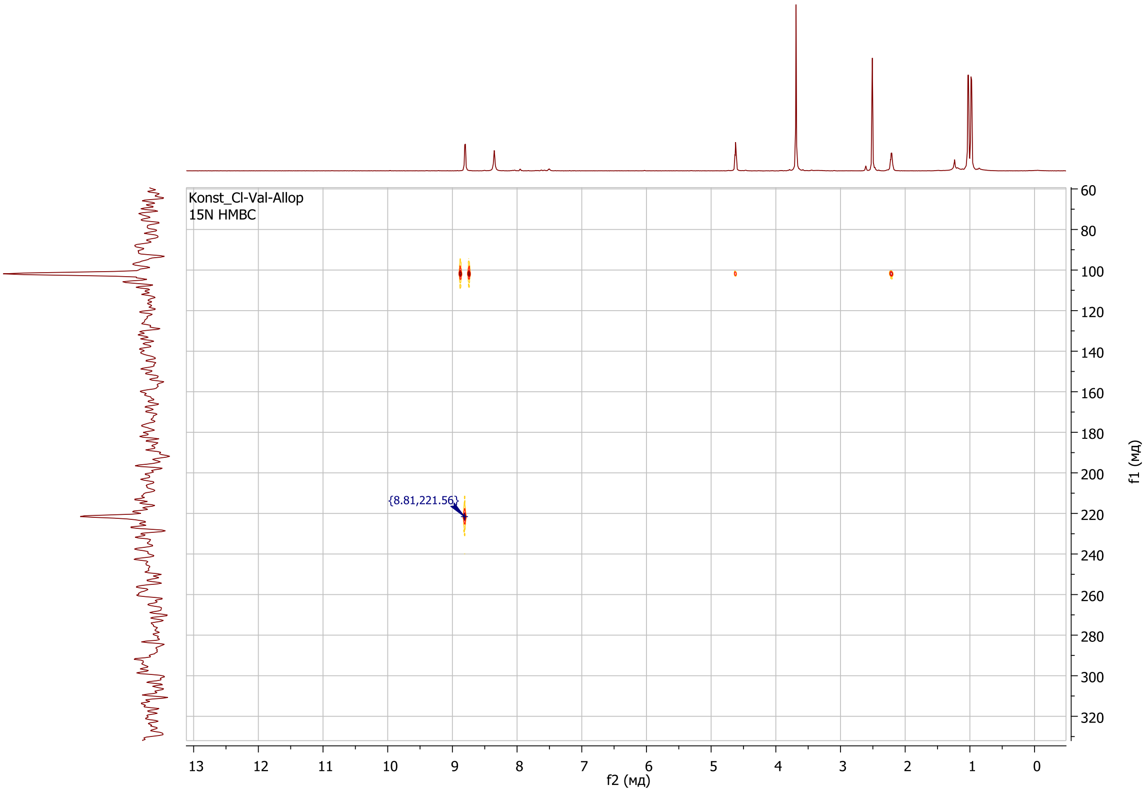

Figure S20. The fragment of ^{15}N HMBC NMR spectrum of 6-chloro-4-(N$^{\alpha}$-L-valinyl)-pyrazolo[3,4-d]pyrimidine methyl ester (**16**)

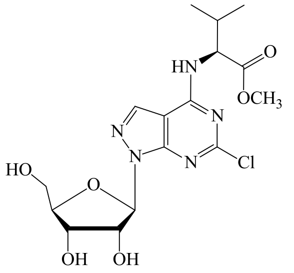 **13**	6-chloro-1-*β*-*D*-ribofuranosyl-4-(N$^{\alpha}$-L-valinyl)-pyrazolo[3,4-d]pyrimidine methyl ester (**13**). ^{1}H NMR (700 MHz, DMSO-d_6, *J*, Hz, 30 °C): δ 9.00 (d, *J* = 7.2 Hz, 1 H, NH-Val), 8.45 (s, 1 H, H3), 6.02 (d, *J* = 4.6 Hz, 1 H, $H_{1'}$), 5.39 (m, 1 H, $OH_{1'}$), 5.15 (m, 1 H, $OH_{2'}$), 4.70 (m, 1 H, $OH_{5'}$), 4.64 (m, 1 H, $^{\alpha}$CH-Val), 4.58 (m, 1 H, $H_{2'}$), 4.20 (m, 1 H, $H_{3'}$), 3.91 (m, 1 H, $H_{4'}$), 3.69 (s, 3 H, OCH_3), 3.56 (m, 1 H, $H_{5'a}$), 3.43 (m, 1 H, $H_{5'b}$), 2.22 (m, 1 H, $^{\beta}$CH-Val), 1.03 (d, *J* = 6.6 Hz, 3 H, CH_3), 0.98 ppm (d, *J* = 6.8 Hz, 3 H, CH_3). ^{13}C NMR (176 MHz, DMSO-d_6, 30 °C): δ 171.53 ($\underline{C}OOCH_3$), 157.03 (C6), 156.60 (C4), 154.72 (C7a), 133.85 (C3), 99.55 (C4a), 88.10 ($C_{1'}$), 85.15 ($C_{4'}$), 72.95 ($C_{2'}$), 70.68 ($C_{3'}$), 62.13 ($C_{5'}$), 58.96 ($^{\alpha}$CH-Val), 51.78 (OCH_3), 30.01 ($\underline{C}(CH_3)_3$), 18.79 ($C(\underline{C}H_3)_3$), 18.68 ppm ($C(\underline{C}H_3)_3$). ^{15}N NMR (71 MHz, DMSO-d_6, 30 °C): δ 305.3 (N2), 222.8 (N5), 199.5 (N1), 102.5 ppm (NH-Val).

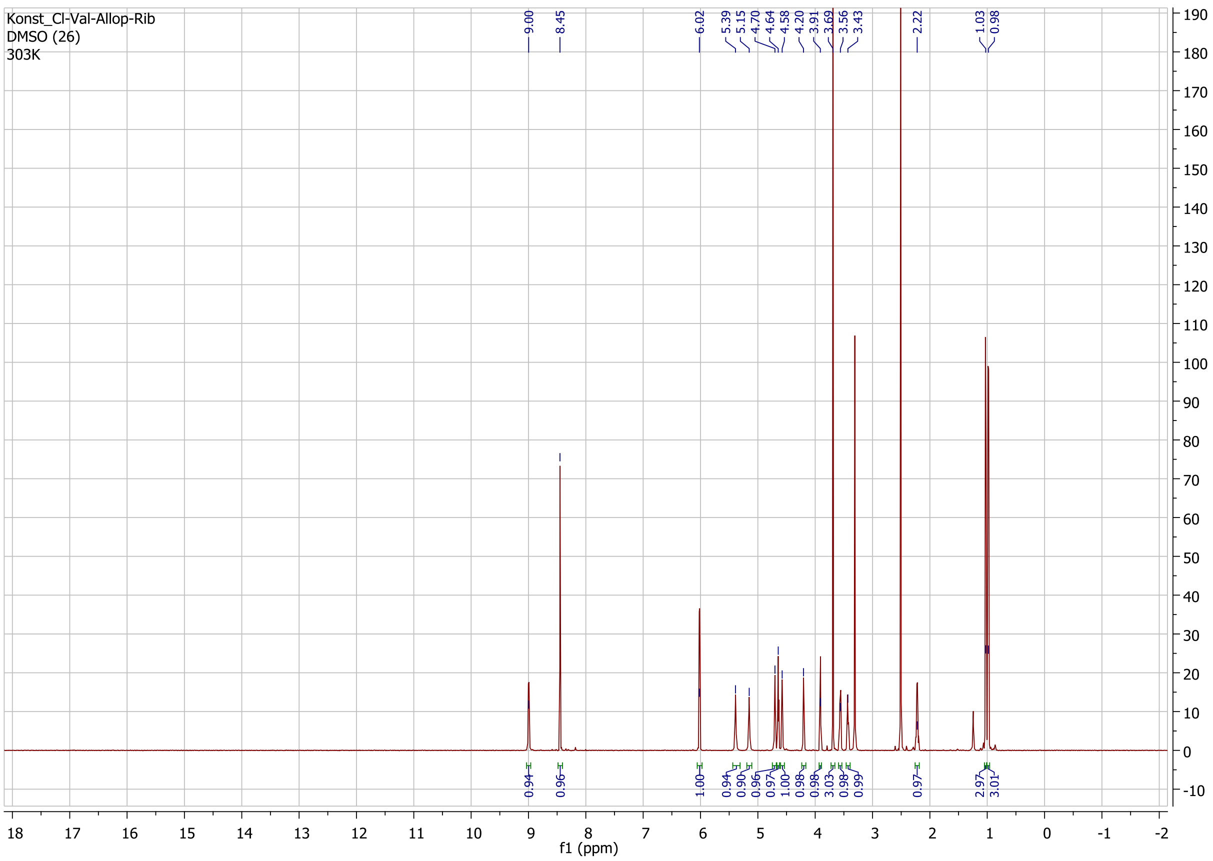

Figure S21. The ^{1}H NMR spectrum of 6-chloro-1-*β*-*D*-ribofuranosyl-4-(N[α]-L-valinyl)-pyrazolo[3,4-d]pyrimidine methyl ester (**13**)

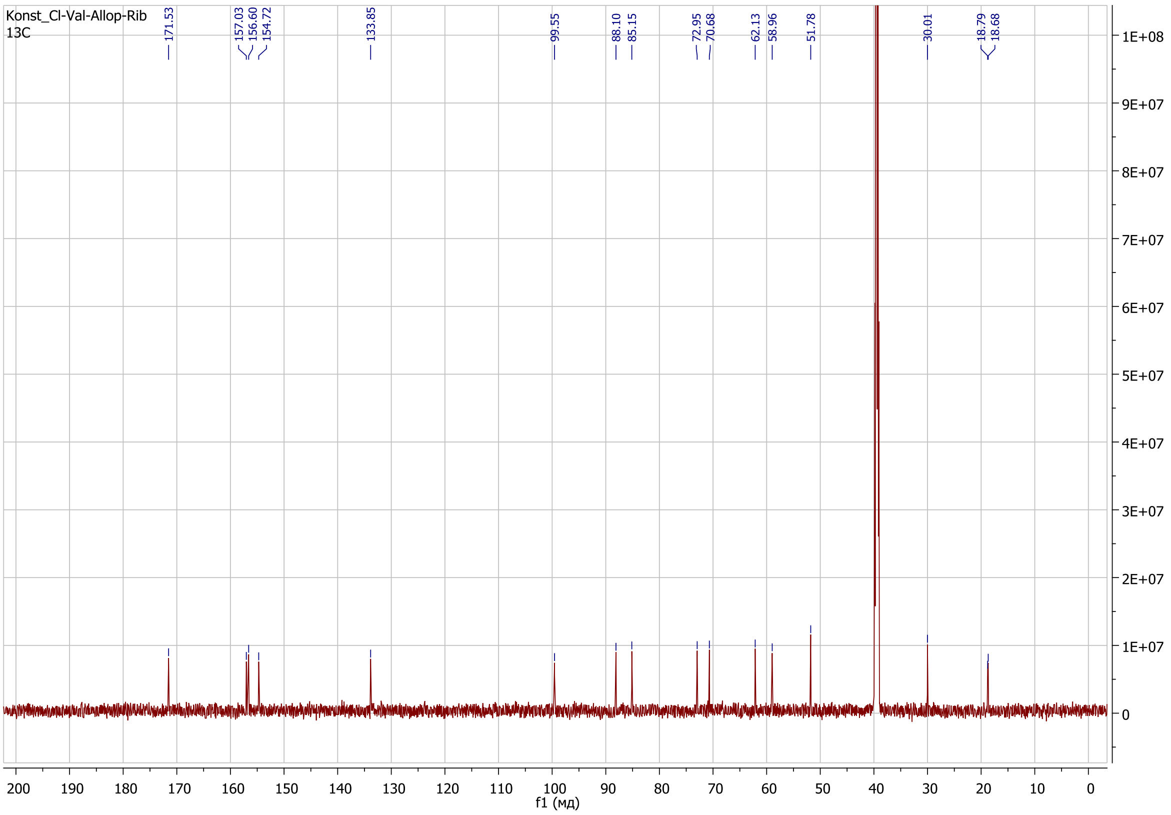

Figure S22. The ^{13}C NMR spectrum of 6-chloro-1-*β*-*D*-ribofuranosyl-4-(N[α]-L-valinyl)-pyrazolo[3,4-d]pyrimidine methyl ester (**13**)

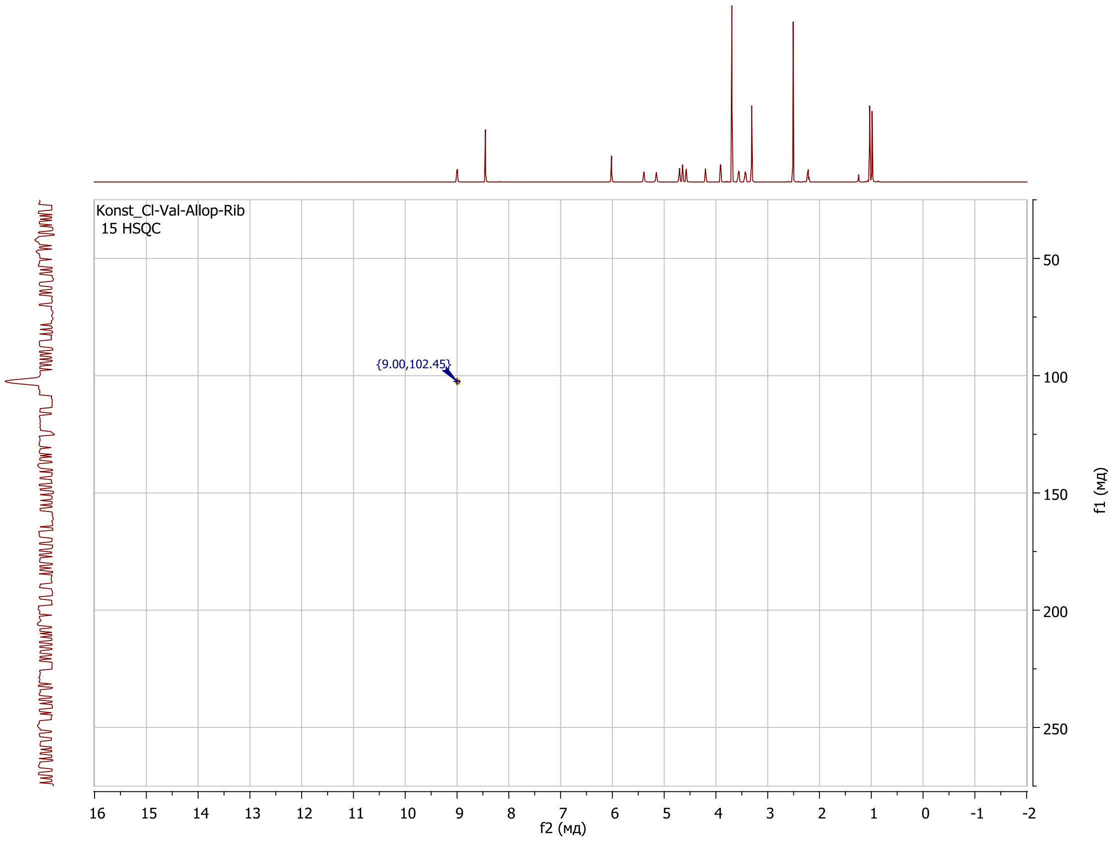

Figure S23. The fragment of ^{15}N HSQC NMR spectrum of 6-chloro-1-*β*-*D*-ribofuranosyl-4-(N$^{\alpha}$-L-valinyl)-pyrazolo[3,4-d]pyrimidine methyl ester (**13**)

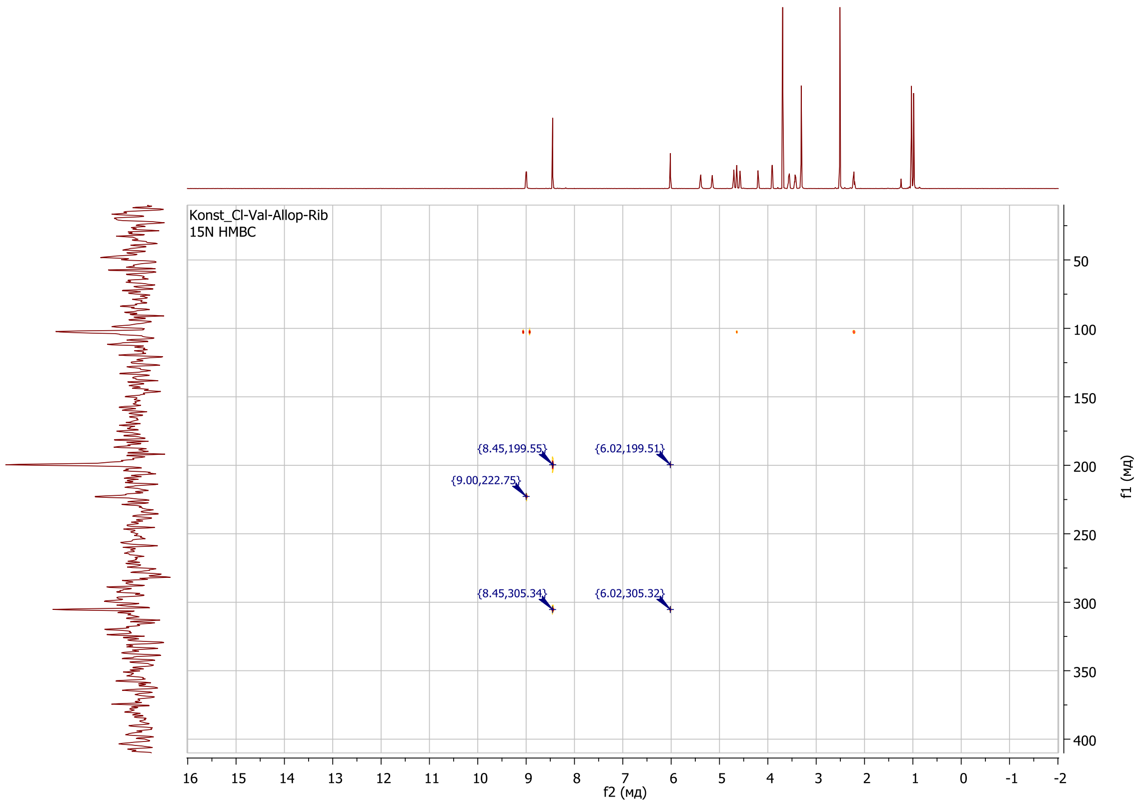

Figure S24. The fragment of ^{15}N HMBC NMR spectrum of 6-chloro-1-*β*-*D*-ribofuranosyl-4-(N$^{\alpha}$-L-valinyl)-pyrazolo[3,4-d]pyrimidine methyl ester (**13**)

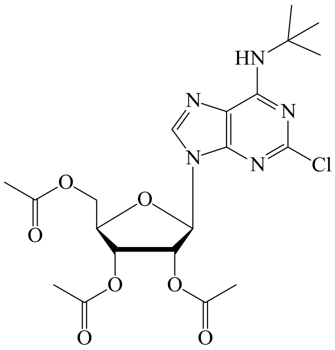<br>**18**	9-(2',3',5'-Tri-*O*-acetyl-*β*-*D*-ribofuranosyl)-2-chloro-6-*tert*-butylamino-purine (**18**).<br><br>^{1}H NMR (700 MHz, DMSO-d_6, J, Hz, 30 °C): δ 8.39 (s, 1 H, H8), 7.57 (br.s, 0.76 H, NH), 6.15 (d, J = 5.5 Hz, 1 H, $H_{1'}$),5.90 (t, J = 5.7 Hz, 1 H, $H_{2'}$), 5.58 (m, 1 H, $H_{3'}$), 4.39 (m, 2 H, $H_{5'a}$ and $H_{4'}$), 4.27 (m, 1 H, $H_{5'b}$), 2.13 (m, 3 H, CH_3CO-3'), 2.05 (m, 3 H, CH_3CO-2'), 2.03 (m, 3 H, CH_3CO-5'), 1.49 ppm (s, 9 H, CH_3). ^{13}C NMR (176 MHz, DMSO-d_6, 30 °C): δ 169.88 (O-$CO^{5'}$), 169.29 (O-$CO^{3'}$), 169.13 (O-$CO^{2'}$), 154.68 (C6), 152.43 (C2), 139.72 (C8), 85.27 ($C_{1'}$), 79.47 ($C_{4'}$), 71.97 ($C_{2'}$), 69.86 ($C_{3'}$), 62.60 ($C_{5'}$), 52.08 ($\underline{C}(CH_3)_3$), 28.46 ($C(\underline{C}H_3)_3$), 20.35 ($\underline{C}H_3$-$CO^{5'}$), 20.24 ($\underline{C}H_3$-$CO^{3'}$), 20.08 ppm ($\underline{C}H_3$-$CO^{2'}$). ^{15}N NMR (71 MHz, DMSO-d_6, 30 °C): δ 243.0 (N7), 167.3 (N9), 114.2 ppm (NH).

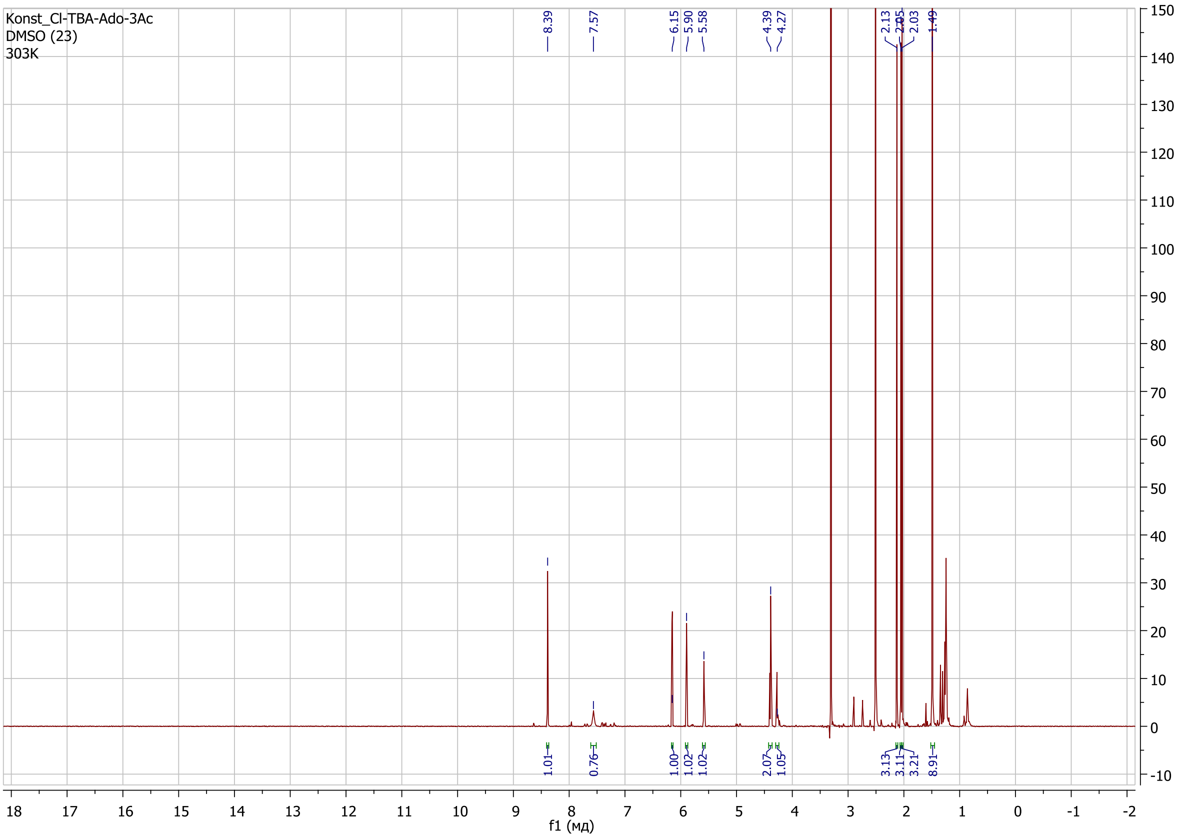

Figure S25. The ^{1}H NMR spectrum of 9-(2',3',5'-tri-*O*-acetyl-*β*-*D*-ribofuranosyl)-2-chloro-6-*tert*-butylamino-purine (**18**)

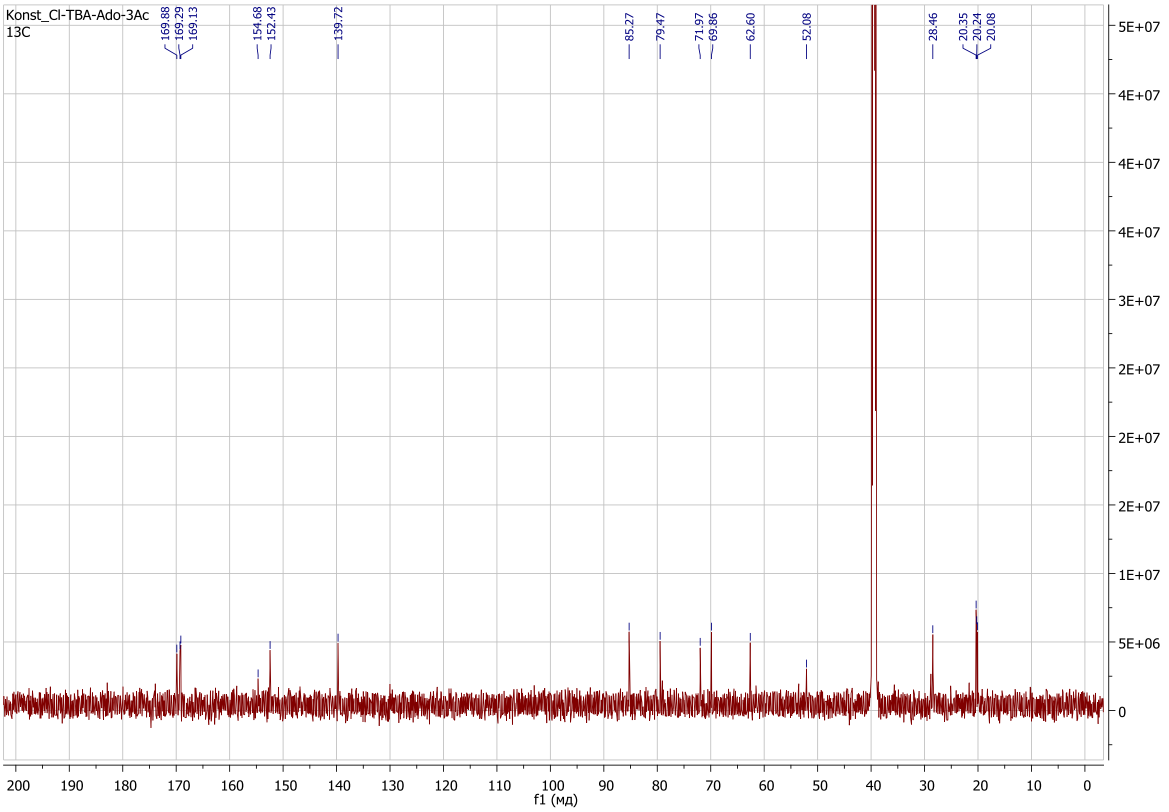

Figure S26. The ^{13}C NMR spectrum of 9-(2',3',5'-tri-*O*-acetyl-*β*-*D*-ribofuranosyl)-2-chloro-6-*tert*-butylamino-purine (**18**)

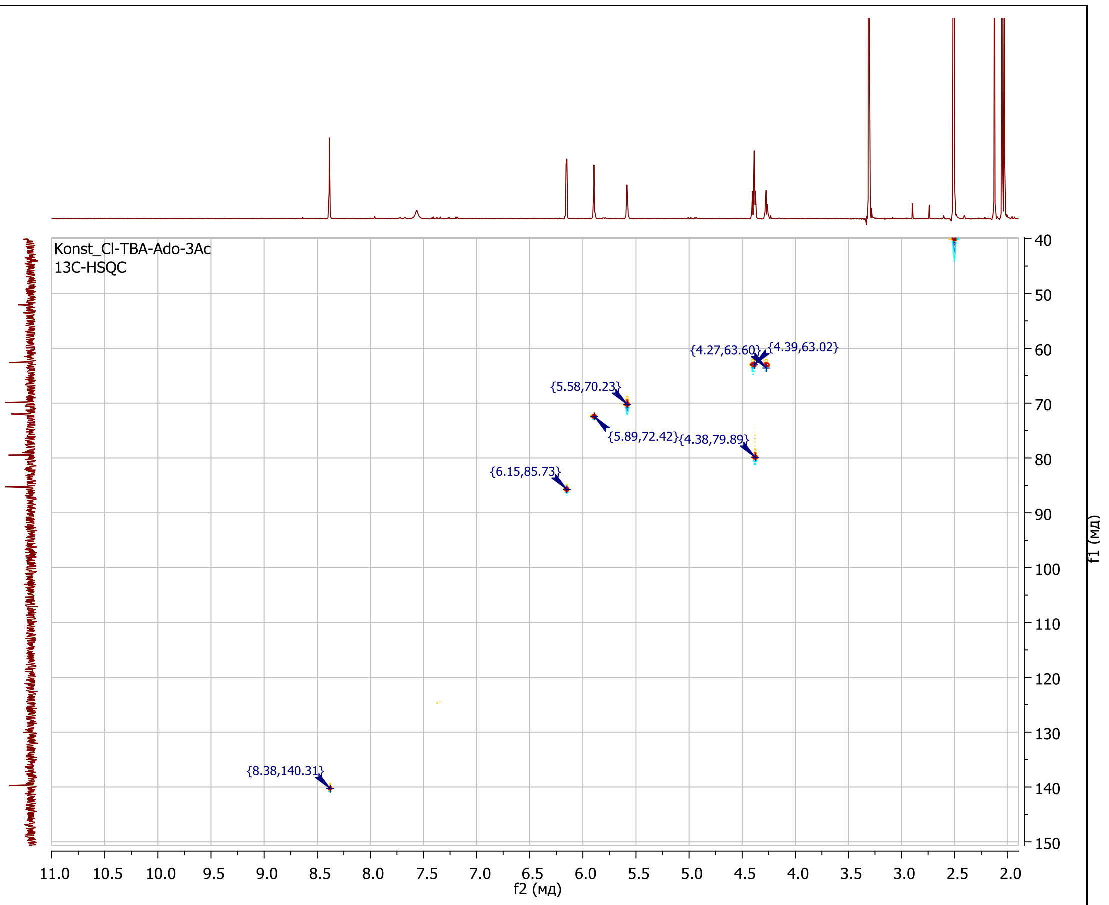

Figure S27. The fragment of ^{13}C HSQC NMR spectrum of 9-(2',3',5'-tri-*O*-acetyl-*β*-*D*-ribofuranosyl)-2-chloro-6-*tert*-butylamino-purine (**18**)

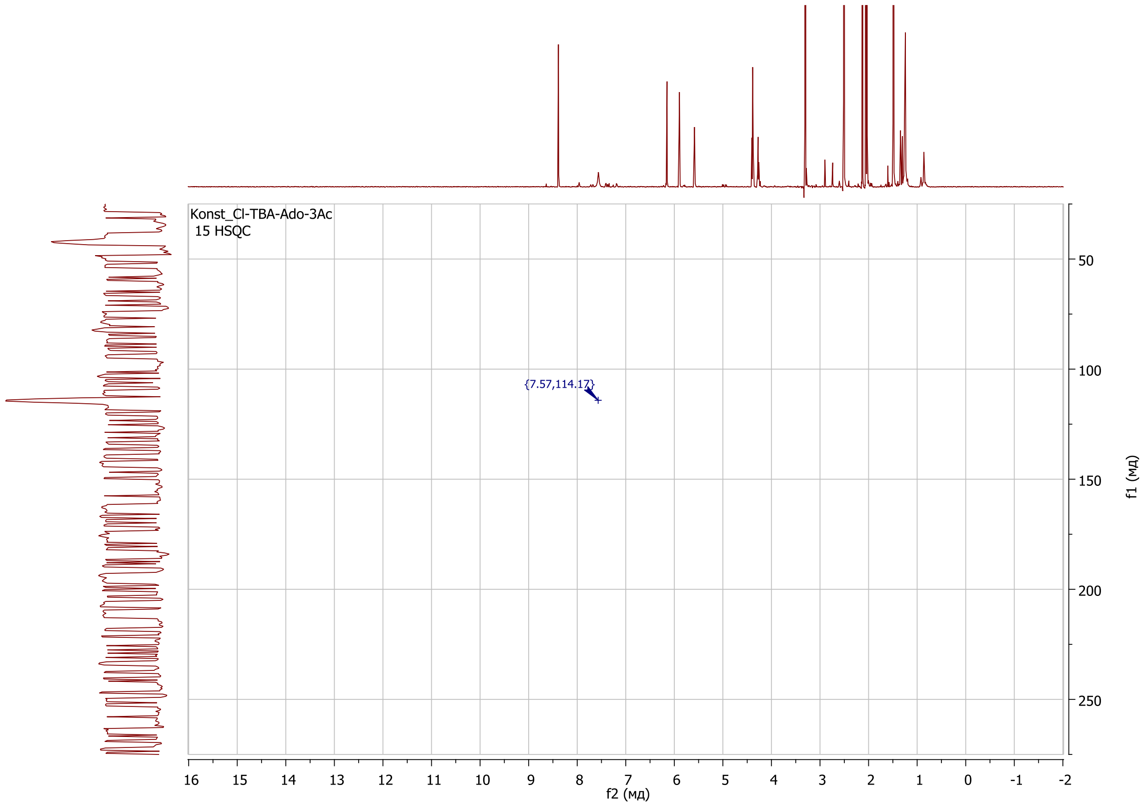

Figure S28. The fragment of ^{15}N HSQC NMR spectrum of 9-(2',3',5'-tri-*O*-acetyl-*β*-*D*-ribofuranosyl)-2-chloro-6-*tert*-butylamino-purine (**18**)

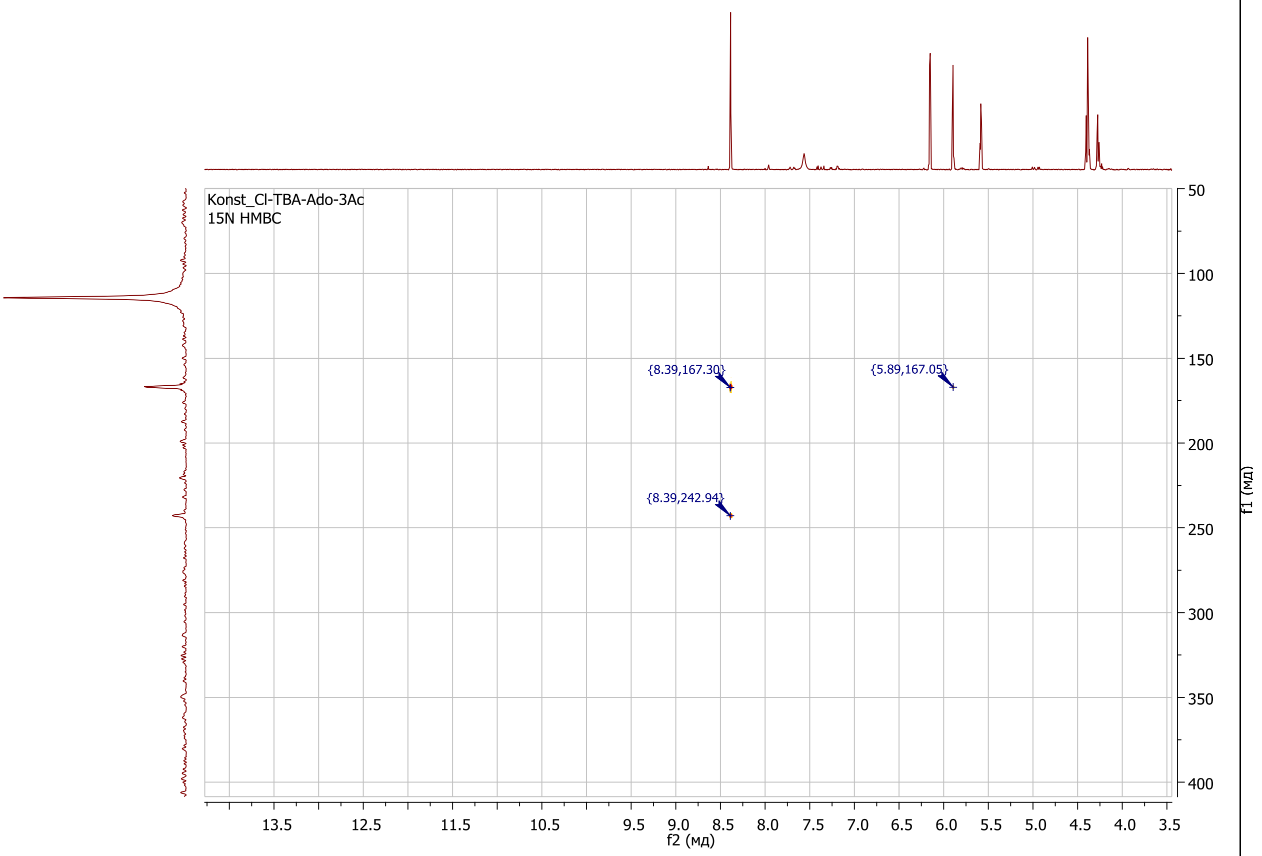

Figure S29. The fragment of ^{15}N HMBC NMR spectrum of 9-(2',3',5'-tri-*O*-acetyl-*β*-*D*-ribofuranosyl)-2-chloro-6-*tert*-butylamino-purine (**18**)

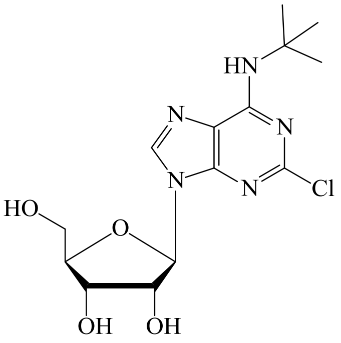

14

9-*β*-*D*-Ribofuranosyl-2-chloro-6-*tert*-butylamino-purine (**14**)

^{1}H NMR (700 MHz, DMSO-d_6, *J*, Hz, 30 °C): δ 8.39 (s, 1 H, H8), 7.46 (br.s, 0.87 H, NH), 5.83 (d, *J* = 5.9 Hz, 1 H, $H_{1'}$), 5.46 (m, 1 H, $OH_{1'}$), 5.19 (m, 1 H, $OH_{2'}$), 5.04 (t, *J* = 5.6 Hz, 1 H, $OH_{5'}$),4.52 (m, 1 H, $H_{2'}$), 4.14 (m, 1 H, $H_{3'}$), 3.95 (m, 1 H, $H_{4'}$), 3.66 (m, 1 H, $H_{5'a}$), 3.57 (m, 1 H, $H_{5'b}$), 1.50 ppm (s, 9 H, CH_3).^{13}C NMR (176 MHz, DMSO-d_6, 30 °C): δ 154.67 (C6), 152.16 (C2), 149.23 (C4), 139.54 (C8), 118.86 (C5), 87.31 ($C_{1'}$), 85.60 ($C_{4'}$), 73.55 ($C_{2'}$),70.25 ($C_{3'}$), 61.26 ($C_{5'}$), 52.02 (NH$\underline{C}$H), 28.52 ppm (CH_3).^{15}N NMR (71 MHz, DMSO-d_6, 30 °C): δ 241.1 (N7), 218.9 (N3), 170.9 (N9), 113.4 ppm (NH).

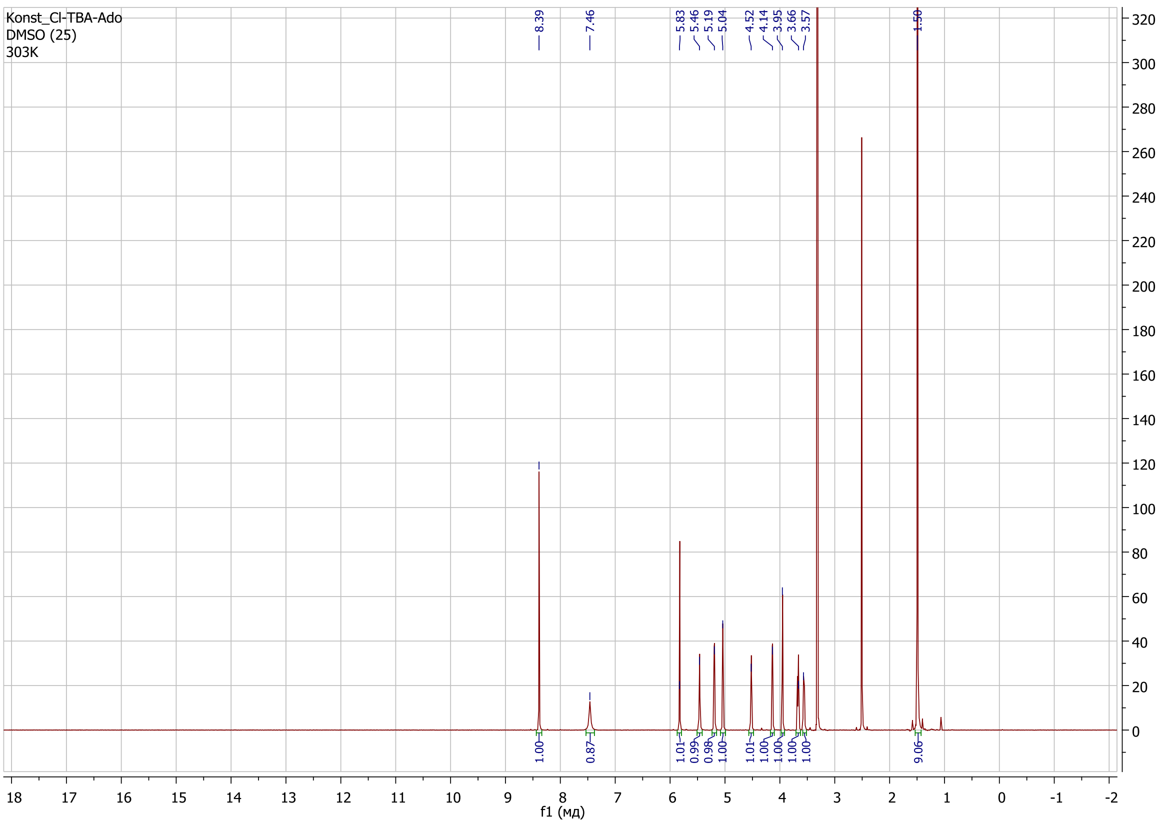

Figure S30. The ^{1}H NMR spectrum of 9-*β*-*D*-ribofuranosyl-2-chloro-6-*tert*-butylamino-purine (**14**)

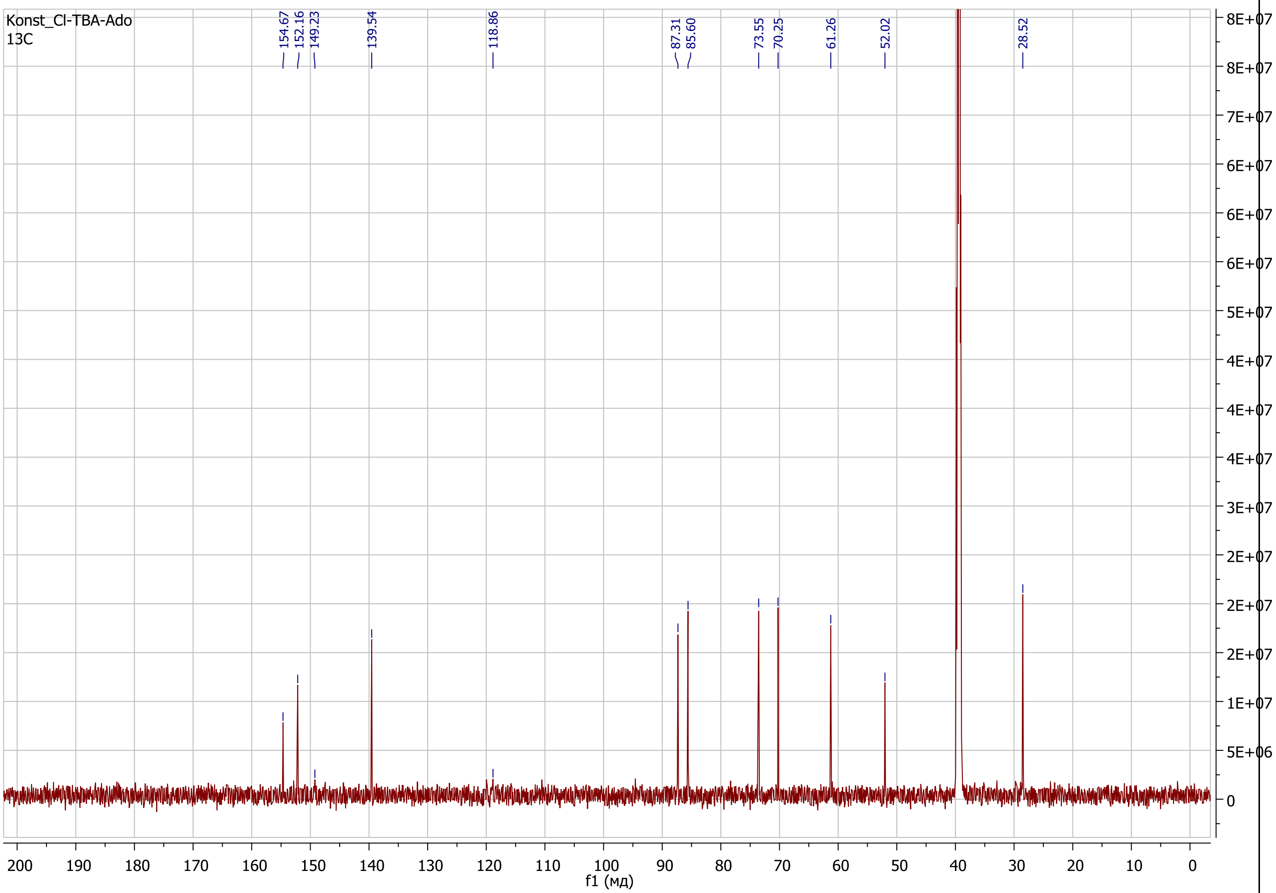

Figure S31. The ^{13}C NMR spectrum of 9-*β*-*D*-ribofuranosyl-2-chloro-6-*tert*-butylamino-purine (**14**)

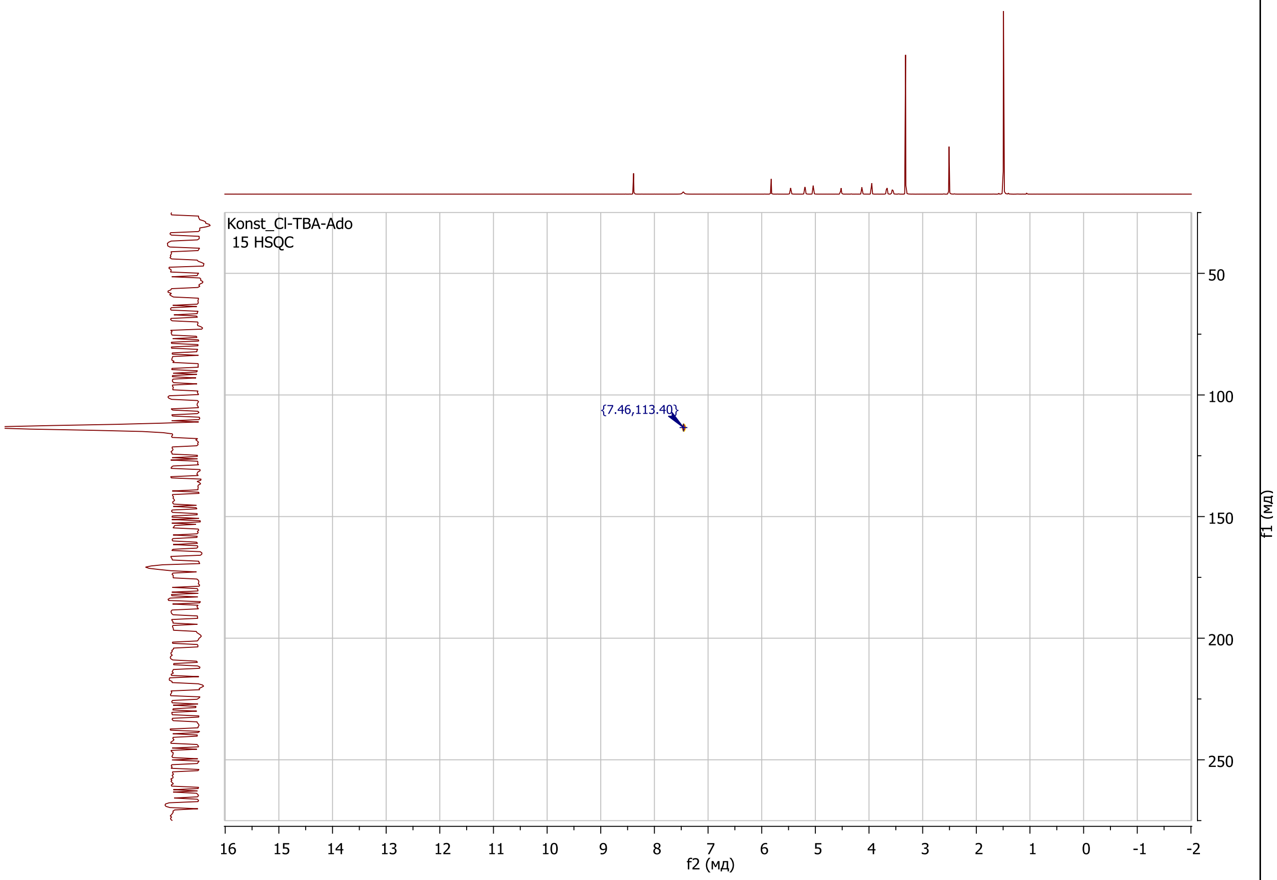

Figure S32. The fragment of ^{15}N HSQC NMR spectrum of 9-*β*-*D*-ribofuranosyl-2-chloro-6-*tert*-butylamino-purine (**14**)

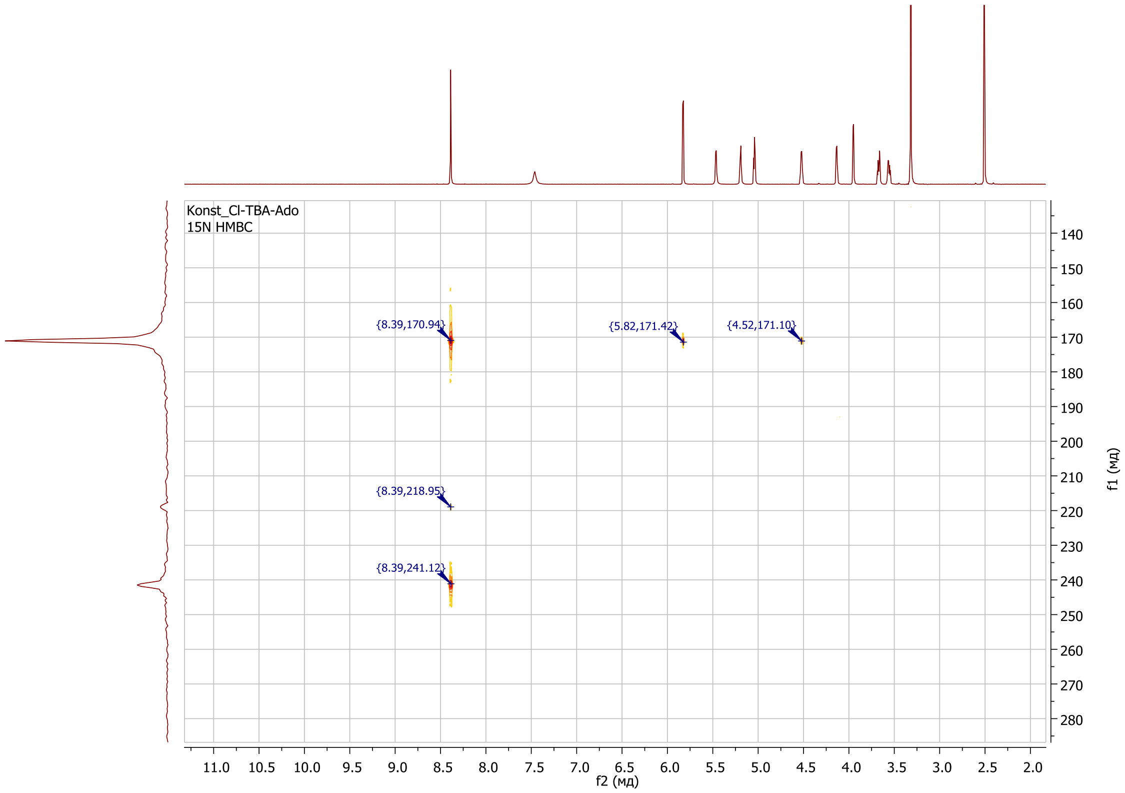

Figure S33. The fragment of ^{15}N HMBC NMR spectrum of 9-*β*-*D*-ribofuranosyl-2-chloro-6-*tert*-butylamino-purine (**14**)

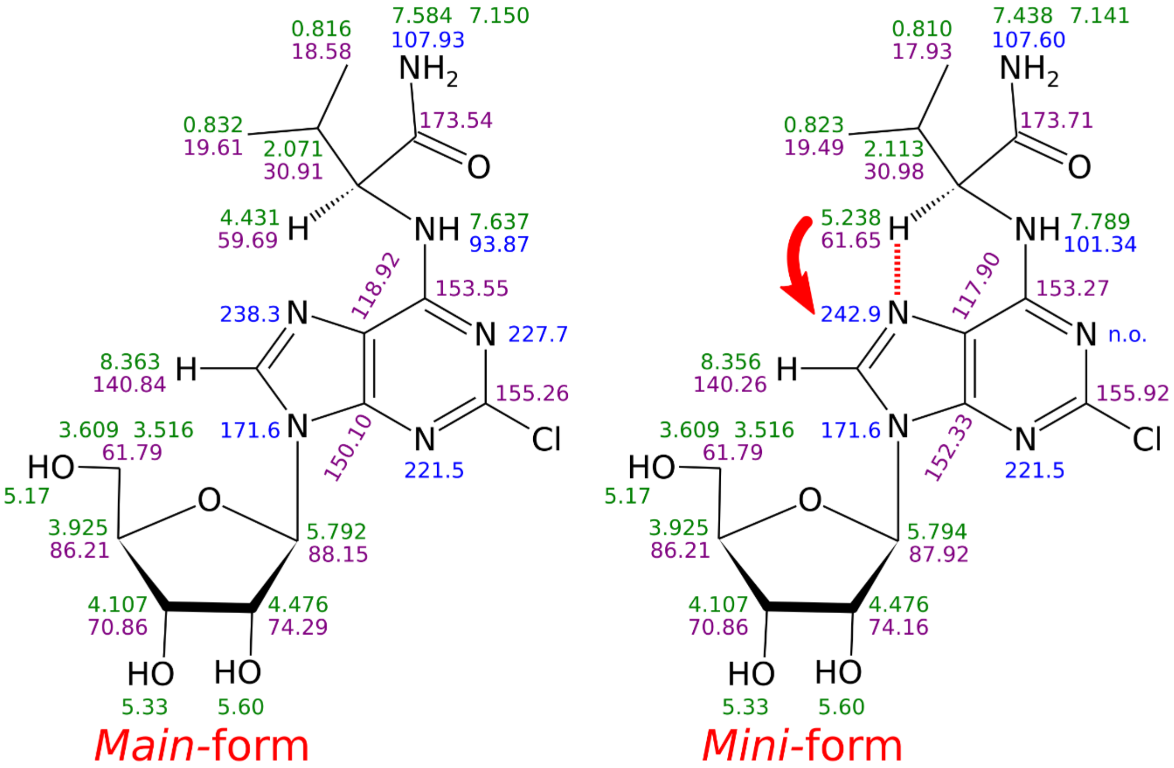

Figure S34. Full assignment of signals of the main and mini-form of compound **3b**.

References

1. Berzina, M. Y.; Eletskaya, B. Z.; Kayushin, A. L.; Dorofeeva, E. V.; Lutonina, O. I.; Fateev, I. V.; Paramonov, A. S.; Kostromina, M. A.; Zayats, E. A.; Abramchik, Y. A.; Maltsev, D. V.; Naumenko L. V.; Taran A. S.; Yakovlev D. S.; Spasov A. A.; Miroshnikov A. I.; Esipov R. S.; Konstantinova I. D. Synthesis of 2-chloropurine ribosides with chiral amino acid amides at C6 and their evaluation as A1 adenosine receptor agonists. *Bioorganic Chemistry* **2022**, *126*, p. 105878. DOI: https://doi.org/10.1016/j.bioorg.2022.105878.
